# SRC Kinase Isoforms Regulate mRNA Splicing during Neural Development

**DOI:** 10.1523/JNEUROSCI.1705-24.2025

**Published:** 2025-08-01

**Authors:** Alastair R. Pizzey, Laura C. West, Samuel J. Elberfeld, Philip A. Lewis, Hannah Walker, Laura Cowell, Katherine Newling, Adam Dowle, Gareth J. O. Evans, Harry V. Isaacs

**Affiliations:** Department of Biology and York Biomedical Research Institute, University of York, Heslington, York YO10 5DD, United Kingdom

**Keywords:** neurogenesis, phosphorylation, SRC, tyrosine kinase, *Xenopus*

## Abstract

Alternative mRNA splicing generates transcriptomic diversity to direct tissue-specific functions. There is a high level of alternative splicing in the brain during embryonic development, but the master regulators of this process are poorly understood. One key splicing event in neuronal differentiation is the inclusion of a microexon in the SH3 domain of the ubiquitous tyrosine kinase, C-SRC, to yield the constitutively active, neural-specific N1-SRC kinase. We previously demonstrated that specific inhibition of N1-SRC in developing *Xenopus* embryos inhibits neurogenesis, but the targets and mode of action of N1-SRC were unknown. In the current study, we screened for N1-SRC SH3 domain interactors, surprisingly finding no unique targets compared with the C-SRC SH3 domain, but rather a subset of binding partners, enriched in splicing regulators. Analysis of public phosphoproteomic data revealed that SRC-dependent phosphorylation of the splicing machinery is widespread and enriched in RNA-binding proteins (RBPs). To investigate whether N1-SRC-dependent regulation of splicing underpins its role in neurogenesis, we undertook long- and short-read RNA-seq analysis of N1-SRC knockdown *Xenopus* embryos. We observed an upregulation of splicing factor expression and aberrant splicing of splicing regulators, principally HNRNPA1 and TRA2A. The affected splice junctions in both genes are in their glycine-rich C-termini, and junctions contain putative binding sites for SFPQ/NONO and FUS RBPs. Both SFPQ and FUS are SRC substrates, suggesting a mechanism by which N1-SRC knockdown leads to mis-splicing of HNRNPA1 and TRA2A. Thus, the neuronal splicing of C-SRC to generate N1-SRC regulates the alternative splicing landscape during neurogenesis.

## Significance Statement

The birth and maturation of neurons during nervous system development is choreographed by transcriptomic reprogramming. The neural-specific N1-SRC splice variant of the SRC nonreceptor tyrosine kinase has roles in neuronal differentiation, but the mechanisms are poorly understood. We show that N1-SRC binds to a subset of SRC interactors that are enriched in RNA-binding proteins (RBPs), suggesting a role of N1-SRC in regulating RNA processing. In support of this, we show altered RNA splicing in N1-SRC knockdown frog embryos. Prominent among genes with altered splicing are HNRNPA1 and TRA2A, which also encode RBPs. Our data support a novel paradigm for the function of SRC family tyrosine kinases as multilevel regulators of the transcriptional/splicing landscape during neural development.

## Introduction

The C-SRC nonreceptor tyrosine kinase is ubiquitously expressed (Ferjentsik et al., 2009; Ortiz et al., 2021). However, in the mammalian nervous system, C-SRC undergoes alternative splicing, yielding neural-specific N1-SRC and N2-SRC proteins, which are identical to C-SRC apart from 6 and 17 amino acid microexon inserts in their respective SH3 domains (Brugge et al., 1985; Pyper and Bolen, 1990). Neural SRC isoforms have higher constitutive kinase activity and altered substrate specificity compared with C-SRC (Dergai et al., 2010; Keenan et al., 2015). While N1-SRC is found widely in vertebrate species, including fish, amphibians, and amniotes, N2-SRC is only present in amniotes (Levy et al., 1987; Martinez et al., 1987; Raulf et al., 1989; Pyper and Bolen, 1990).

N1-SRC is highly expressed in neural development in vivo (Wiestler and Walter, 1988) and during the in vitro differentiation of neuronal cell lines (Lynch et al., 1986; Cartwright et al., 1987; Matsunaga et al., 1993). In addition, high N-SRC expression is linked with a positive prognosis for neuroblastoma tumors through their propensity to differentiate. Thus, the substrate specificity and high constitutive activity of N1-SRC confers distinct functions from C-SRC in neuronal differentiation. Indeed, we have shown that overexpression of N1-SRC, but not C-SRC, is sufficient to elicit neurite-like outgrowths in cultured fibroblasts (Keenan et al., 2017; Lewis et al., 2017). Overexpression of N1-SRC and C-SRC also has differential effects on axonogenesis in developing retinal precursors (Worley et al., 1997). Conversely, knockdown of N1-SRC expression in cultured rat hippocampal neurons inhibits neurite growth (Keenan et al., 2017). Furthermore, a specific knockdown of N1-SRC in developing *Xenopus* embryos, which does not affect C-SRC expression, reduces the differentiation of motor and inter- and sensory neurons at open neural plate stages, and in later development, embryos exhibit abnormal touch responses (Lewis et al., 2017). These data point to a role of N1-SRC earlier in neurogenesis that is distinct from its established roles in neuronal maturation; however, the mechanisms are unknown.

In this study, we used proteomic and transcriptomic approaches to shed light on the neuronal binding partners of the N1-SRC SH3 domain and the impact of N1-SRC knockdown on the transcriptional landscape during neurogenesis. We found that the N1-SRC SH3 domain does not have unique binding partners but binds to a subset of C-SRC SH3 ligands, notably enriched in RNA-binding proteins (RBPs). Consistent with a role of N1-SRC in regulating RBPs, knockdown of N1-SRC in *Xenopus* embryos upregulated the expression of a cohort of RNA processing genes and perturbed mRNA splicing. The splicing factors HNRNPA1 and TRA2A were prominent among genes with altered splicing. The splice junctions of *Hnrnpa1* and *Tra2a* affected by N1-Src knockdown contain multiple consensus binding sites for RBPs that are SRC substrates, including SFPQ. We propose that N1-SRC phosphorylation might coordinate splicing during neuronal development by regulating the phosphorylation of specific RBPs present at splice sites.

## Materials and Methods

### SRC SH3 pulldown

Recombinant GST-C-Src SH3 and GST-N1-Src SH3 domains were expressed in BL21 *E. coli* and purified on glutathione Sepharose resin according to a previously published protocol (Keenan et al., 2015). The 12.5 µM GST or GST-Src SH3 proteins immobilized on glutathione Sepharose resin were incubated with 2 mg P0 rat brain lysate protein for 2 h at 4°C with agitation. The resin was pelleted by centrifugation for 2 min at 16,000 × *g* at 4°C. The supernatant was removed, and the resin was transferred to spin-X centrifuge columns (Corning) that were pre-equilibrated with PBS. The resin was then washed 6–8 times in a 800 μl wash buffer for 1 min at 16,000 × *g* at 4°C, before a 10 min incubation with a Laemmli sample buffer, and subsequent elution via centrifugation for 10 min at 16,000 × *g*. Following SDS-PAGE gel electrophoresis of the GST-C- and N1-Src SH3 domain pulldowns, the gel was excised into two fractions ∼<40 and >40 kDa to separate the GST-SH3 domain bait and the remainder, respectively. The gel pieces were then further subdivided into 1 mm pieces and washed twice with 25 mM ammonium bicarbonate in 50% (v/v) acetonitrile for 20 min and then once with acetonitrile for 5 min, followed by drying for 20 min under a vacuum. The gel was then incubated with 10 mM DTT in 100 mM ammonium bicarbonate for 1 h at 56°C. The supernatant was removed, and the gel was incubated with 50 mM iodoacetamide in 100 mM ammonium bicarbonate in the dark for 30 min at room temperature. The supernatant was removed, and the gel was washed in 100 mM ammonium bicarbonate for 15 min, then with 25 mM ammonium bicarbonate in 50% acetonitrile for 15 min, and acetonitrile for 5 min. The supernatant was removed, and the gel was dried for 20 min under a vacuum. The gel samples were incubated with trypsin at 25 μg/ml in 25 mM ammonium bicarbonate overnight at 37°C. The following day, the supernatant containing the digested peptides was retained, and any remaining peptide was extracted by incubation with 50% acetonitrile for 15 min; this was repeated twice, and the extracts were pooled with the supernatant. The combined supernatant was then dried under a vacuum, and the peptides were reconstituted in 0.1% trifluoroacetic acid for analysis by LC-MS/MS.

### LC-MS/MS analysis

Samples were loaded onto an UltiMate 3000 RSLCnano HPLC system equipped with a PepMap 100 Å C18, 5 μm trap column (300 μm × 5 mm), and a PepMap, 2 μm, 100 Å, C18 EasyNano nanocapillary column (75 m × 500 mm). The trap wash solvent was aqueous 0.05% (v/v) trifluoroacetic acid, and the trapping flow rate was 15 μl/min. The trap was washed for 3 min before switching flow to the capillary column. Separation used gradient elution of two solvents: Solvent A, aqueous 1% (v/v) formic acid, and Solvent B, aqueous 80% (v/v) acetonitrile containing 1% (v/v) formic acid. The flow rate for the capillary column was 300 nl/min, and the column temperature was 30°C. The linear multistep gradient profile was 3–10% B over 7 min, 10–35% B over 30 min, and 35–99% B over 5 min and then proceeded to wash with 99% Solvent B for 4 min. The column was returned to initial conditions and re-equilibrated for 15 min before subsequent injections. The nanoLC system was interfaced with an Orbitrap Fusion hybrid mass spectrometer with an EasyNano ionization source. Positive ESI-MS and MS2 spectra were acquired using the Xcalibur software (version 4.0). Instrument source settings were as follows: ion spray voltage, 1,900 V; sweep gas, 0 arb; and ion transfer tube temperature, 275°C. MS1 spectra were acquired in the Orbitrap with the following: 120,000 resolution; scan range, m/z 375–1,500; AGC target, 4e5; and max fill time, 100 ms. Data-dependent acquisition was performed in a top-speed mode using a fixed 1 s cycle, selecting the most intense precursors with charge states 2–5. Easy-IC was used for internal calibration. Dynamic exclusion was performed for 50 s postprecursor selection, and a minimum threshold for fragmentation was set at 5,000. MS2 spectra were acquired in the linear ion trap with the following: scan rate, turbo; quadrupole isolation, 1.6 m/z; activation type, HCD; activation energy, 32%; AGC target, 5e3; first mass, 110 m/z; and max fill time, 100 ms. Acquisitions were arranged by Xcalibur to inject ions for all available parallelizable time. The proteomics analysis pipeline is shown in Extended Data Fig. 1-1.

### Bioinformatic analysis of tyrosine phosphorylation of the splicing machinery

Datasets representing all phosphosites (“phosphorylation_site_dataset”) and validated phosphosites (“kinase_substrate_dataset”) were downloaded from the PhosphoSitePlus database (Hornbeck et al., 2015). The phosphorylation_site_dataset was filtered to include only phosphosites observed by low-throughput experiments (LT_LIT) or three or more high-throughput experiments (MS_LIT and MS_CST). The resulting dataset was then filtered by residue (MOD_RSD) to provide serine, threonine, and tyrosine phosphosite datasets. The kinase_substrate_dataset was filtered to obtain known Src substrate tyrosine phosphosites. A list of splicing machinery proteins was obtained from Spliceosome Database (Cvitkovic and Jurica, 2013). The human dataset, comprising 1,111 proteins, was filtered by function (class/family) to yield 317 proteins directly involved in splicing or its regulation. The tyrosine phosphosite dataset and the Src substrate dataset were compared with the splicing machinery dataset to identify all tyrosine phosphosites and those identified as Src substrates in the splicing machinery. The proportion of known and predicted Src phosphosites in each subclass of the splicing machinery was calculated and visualized by a heatmap (Morpheus, https://software.broadinstitute.org/morpheus). All data tidying, filtering, and merging were performed in R.

### In vitro kinase assay

In vitro kinase assays performed on SFPQ GST-fusion peptides with His-Δ80-N1-Src as previously described (Keenan et al., 2015). Briefly, sequences corresponding to regions surrounding Src phosphosites of rat SFPQ (480/482 and 519) were subcloned into pGEX-4T-1 and expressed and purified from BL21 *E .coli*. A 10 µg of substrate GST-peptide, 1 µg N1-Src, and 500 uM ATP were prepared in kinase reaction buffer (100 mM Tris, 10 mM MgCl_2_), pH 7.5, to a final volume of 50 µl. An ideal Src substrate motif (YEEI) peptide fused to GST was used as a positive control and GST alone as a negative control. Reactions were incubated at 30°C for 3 h, terminated by the addition of a 2× SDS-sample buffer, and separated by SDS-PAGE, transferred to PVDF membrane and immunoblotted for phosphotyrosine (PY20; BD Biosciences). Immunoreactivity was visualized by incubation of immunoblots with enhanced chemiluminescence reagent (Millipore) and exposure with an iBRIGHT imaging system (Invitrogen). To ensure equal protein loading, we stained the samples with Coomassie gel stain to detect the substrates.

### Embryological methods

All work involving animals was approved by the University of York Animal Welfare and Ethical Review Body and performed under UK Home Office legislation (project license POF 245295). *Xenopus tropicalis* and *Xenopus laevis* embryo culture methods were as previously described (Khokha et al., 2005; Tindall et al., 2007; Winterbottom et al., 2010). The sequences of the *Xenopus* N1-SRC splice-blocking antisense morpholino oligos (AMOs; GeneTools) were as previously reported (Lewis et al., 2017).

### mRNA synthesis

Open reading frames encoding C-terminal FLAG-tag wild–type rat C- and N1-SRC were amplified with Phusion DNA polymerase and subcloned into the Xho1 site of pCS2+. The C-terminal FLAG-tag *Xenopus laevis* N1-Src open reading frame was amplified and subcloned into the Xba1 site of pCS2+. The rat CS+ SRC constructs were linearized with Asp718, and *Xenopus* N1-Src was linearized with Not1. Synthetic mRNA was synthesized using the Message mMachine SP6 Transcription Kit (Invitrogen).

### Western blot analysis of embryos

Five embryos were flash frozen at Neurula Stage 17 and lysed in 2× denaturing sample buffer prior to electrophoresis. Samples were separated by SDS-PAGE, transferred onto PVDF membrane and probed with relevant primary antibodies: mouse anti-FLAG(M2) (Sigma-Aldrich) primary antibody (1:1,000) and rabbit anti-SRC pY416 (Cell Signaling Technology). Secondary HRP-conjugated antibodies (Sigma-Aldrich) were anti-mouse (1:5,000) and anti-rabbit (1:5,000). Signals were visualized using Immobilon Chemiluminescent HRP substrate and an autoradiographic film.

### RNA extraction and semiquantitative RT-PCR

Total RNA was extracted from flash frozen embryos using Tri-Reagent (Sigma-Aldrich) according to the manufacturer's instructions. An additional RNA precipitation step was carried out with 7.5 M LiCl/50 mM EDTA at −80°C overnight. First-strand cDNA was synthesized using 0.5 μg of total RNA, random hexamer primers, and SuperScript IV Reverse Transcriptase (Thermo Fisher Scientific). Targets were amplified using PCR Master Mix (Promega) and the primers listed in Table 1. Gel pictures presented are representative of at least three biological repeats.

**Table 1.** RT-PCR primers

Primer	Sequence (5′-3′)	Annealing temperature (°C)	Amplicon size (bp)
*n1-src* forward *n1-src* reverse	ACTGTGACCTGACGCCTTTT CCTCATGTCAGGTCTCGTGTT	50	160
*rpl8* forward *rpl8* reverse	GGGCTRTCGACTTYGCTGA ATACGACCACCWCCAGCAA	50	436
*sox3* forward *sox3* reverse	CCAGAGGATAGACACTTAT CTACTCTGAAGGGAAGAA	45	414
*irx1* forward *irx1* reverse	GAGGAAGAGGATGAGAAA GAGGAAGAGGATGAGAAA	45	677
*neurod1* forward *neurod1* reverse	CTCTCTCCGAGATTCTAC GGCACTCATTACTCTTC	50	584
*hnrnpa1* forward *hnrnpa1* reverse	GTTATGGTGGAGATGGCTACAA CCACCATAATTGCCACCTTTC	50	308

### RNA-seq library preparation

RNA was extracted from Midneurula Stage 16 sibling *Xenopus tropicalis* embryos injected with 10 ng AMO A + D or equal concentration standard control morpholino. Flash frozen embryos were homogenized in Tri-Reagent (Sigma-Aldrich) as per the manufacturer's instructions. After extraction, RNA was purified using RNA Clean & Concentrator-25 (Zymo Research) as per manufacturer's instructions. RNA integrity was measured on Agilent 2100 Bioanalyzer. Samples with low RNA Integrity Numbers were further purified via 2.5 M lithium chloride precipitation overnight at −20°C. Library preparation was carried out by the staff at the University of York Bioscience Technology Facility (BTF). Poly(A) mRNA was isolated from total RNA by the NEBNext Poly(A) mRNA Magnetic Isolation Module, and the NEBNext RNA Ultra II Directional RNA Library Prep Kit was used to generate the cDNA library. Transcripts were sequenced on the Illumina HiSeq 3000 machine at the University of Leeds. Illumina sequencing resulted in ∼35–50 million reads per sample. Long-read (LR) sequencing cDNA libraries were generated using the Oxford Nanopore PCR-cDNA Sequencing Kit and were sequenced on a PromethION machine at the University of York (BTF). Sequencing data files have been submitted to the GEO database with accession number GSE290070.

### Assembly of Nanopore LR transcriptome

HISAT2 (http://www.ccb.jhu.edu/software/hisat2; Kim et al., 2019) and StringTie (https://ccb.jhu.edu/software/stringtie/index.shtml; Pertea et al., 2015) were used for Nanopore LR transcriptome assembly. Transcripts were merged back to the reference annotation with gffcompare to link gene names to the StringTie assembled transcripts (https://github.com/gpertea/gffcompare; Pertea and Pertea, 2020).

### Gene-level differential expression analysis

Illumina reads were aligned to the *Xenopus tropicalis* reference transcriptome (version 9.1) with Salmon, which was used to aggregate transcript expression to estimate gene expression levels (http://salmon.readthedocs.io; Patro et al., 2017). Differential gene expression in control versus N1-SRC morpholino-injected (morphant) embryos was analyzed by Sleuth (Version 0.30; http://pachterlab.github.io/sleuth/; Pimentel et al., 2017). Genes with group mean expression values of <1 transcript per million (TPM) for control and N1-SRC morphant embryos were removed from the analysis. Genes with *q* values/false discovery rates (FDRs) ≤ 0.05 were considered to be differentially expressed in control and N1-SRC morphant embryos. Significantly up- and downregulated genes were further categorized using the criteria, *b* values (log2 [effect size]) >0 and *b* values <0, respectively.

### Transcript-level differential expression analysis

Illumina reads were aligned to the *Xenopus tropicalis* Nanopore LR transcriptome assembly, and transcript expression levels were estimated with Salmon. Differential gene expression in control versus N1-SRC morphants was analyzed by Sleuth using the same filtering criteria described above.

### Identification of differential splicing events

Illumina short reads (SR) were aligned to the Nanopore LR transcriptome, and the alignments were used as input for IsoformSwitchAnalyzeR (https://www.bioconductor.org/packages/release/bioc/html/IsoformSwitchAnalyzeR.htm; Vitting-Seerup and Sandelin, 2019), which identified pairs of isoforms with opposite changes in usage between the two experimental conditions, where at least one of the changes is significant (*Q* < 0.1). Transcripts with significant changes in usage between control and *n1-src* morphants were manually inspected and confirmed. Statistical comparisons for gene switching and differential expression of individual transcripts (corrected for multiple testing) were calculated by IsoformSwitchAnalyzeR using Cuffdiff. IsoformSwitchAnalyzeR does not output the class splicing event or its genomic coordinates. Therefore, all validated switching events between pairs of transcripts/isoforms were submitted to the Alternative Splicing transcriptional landscape visualization tool (AStalavista; Foissac and Sammeth, 2007) using our LR RNA-seq reference *Xenopus tropicalis* transcriptome to obtain genomic coordinates for the splice site RBP site analysis. Alternative transcription start or termination events were not included in the analysis. The RNA-seq analysis pipeline is shown in Extended Data Fig. 3-1.

### Identification of enriched RNA-binding motifs

One hundred base sequences upstream (IR1 and IR3) and downstream (IR2 and IR4) of affected splice junctions were scanned for consensus RBP binding sites using the PWM log odds algorithm (threshold 6) using the CISBP-RNA web interface (http://cisbp-rna.ccbr.utoronto.ca/; Ray et al., 2013).

### PANTHER Gene Ontology (GO) analysis

GO analysis was carried out using the PANTHER Classification System version 17 (Mi et al., 2021). Differentially expressed gene lists were submitted for analysis via the web interface with *Xenopus tropicali*s selected as input and reference genome (http://pantherdb.org/). Multiple entries for individual genes in differentially expressed transcript lists were removed, and unique gene lists were submitted for analysis. Statistical overrepresentation for PANTHER GO complete biological process terms was calculated using Fisher's exact test with FDR correction. Additional stringent filtering criteria were used on the enriched GO term output from PANTHER (client input ≥0, enrichment ≥2, and FDR ≤0.05).

### STRING association network analysis

STRING networks, indicating functional and physical protein associations, were constructed based on lists of unique genes using the STRING-db online tool (https://version-11-5.string-db.org/) using a minimum required interaction score of 0.4. Text mining, experiments, databases, coexpression, neighborhood, gene fusion, and co-occurrence data were used for construction of the networks (Szklarczyk et al., 2019 , 2021). Figures were prepared by exporting data into Cytoscape 3.10 (https://cytoscape.org/; Shannon et al., 2003) and preparing networks with edge thickness indicating strength of evidence.

## Results

### The N1-SRC SH3 domain binds a subset of C-SRC SH3 ligands enriched in regulators of mRNA metabolism

Alternative splicing of a microexon resulting in a small insertion into the N1-SRC SH3 domain is the basis for the different biological functions of C-SRC and N1-SRC. The SH3 domain is a key protein–protein interaction module, and in order to gain insights into the mechanisms of N1-SRC function, we compared the interactomes of the C-SRC and N1-SRC SH3 domains.

As N1-SRC is important for neural development and is highly expressed early in development of the rodent forebrain, but not significantly in the cerebellum (Wiestler and Walter, 1988), we performed pulldowns from neonatal rat forebrain lysates using recombinant GST, GST-N1-SRC SH3, or GST-C-SRC SH3 fusion proteins as bait. Interacting proteins were identified by LC-MS/MS, with relative quantification between samples by spectral counting (Extended Data Figs. 1-1, 1-2). The C-SRC SH3 domain had 176 significant binding partners compared with the GST control. Thirty-three ligands were found to significantly associate with the N1-SRC SH3 domain, none of which are unique to N1-SRC. Thus, N1-SRC SH3 interactors are a subset of the larger cohort of C-SRC interactors (Extended Data Fig. 1-2).

**Figure 1.**
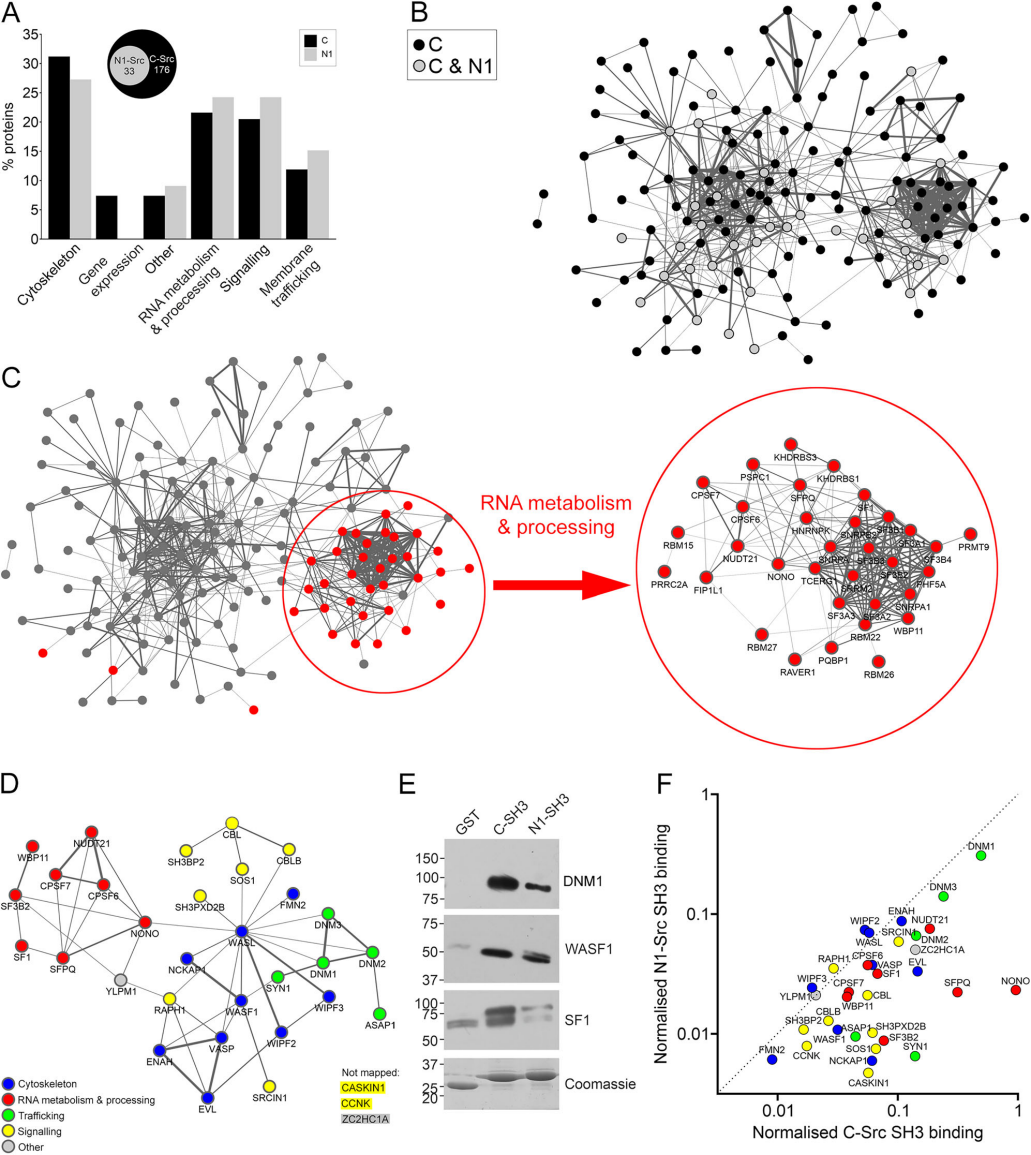
The N1-SRC SH3 domain binds a subset of C-SRC SH3 ligands enriched in regulators of mRNA metabolism. ***A***, The plot showing the relative proportion of protein functional groups for the C- and N1-SRC SH3-specific binding partners. The Venn diagram highlights that the 33 N1-SRC SH3 binding partners are a subset of the C-SRC SH3 binders. ***B***, A STRING network of the significant 176 C- and N1-SRC SH3 domain binding partners identified by LC-MS/MS. Nodes shaded in black represent proteins identified as binding only to the C-SRC SH3 domain. Nodes shaded in gray bind to both C-SRC and N1-SRC SH3 domains. ***C***, A STRING network of the significant C- and N1-SRC SH3 domain binding partners identified by LC-MS/MS. Nodes colored in red represent proteins involved in RNA metabolism and are presented as a labeled network in the enlarged circled inset. ***D***, STRING network of the N1-SRC–specific binding partners colored by the functional group. ***E***, Immunoblots of the GST pulldown elutions with antibodies raised to representative SH3 domain binding partners (dynamin I, WASF1, and SF1). Bottom panel, Coomassie staining of the gel to confirm equal loading of the bait GST-fusion proteins. ***F***, A plot of the relative abundance (calculated by spectral counts) of the 33 N1-SRC SH3 interactors against their C-SRC SH3 binding. (See Extended Data Fig. 1-1 for a schematic of the proteomics pipeline employed, Extended Data Fig. 1-2 for the mass spectrometry data, Extended Data Figs. 1-3 and 1-4 for functional annotation of C-SRC and N1-SRC SH3 domain interacting proteins.)

PANTHER protein and UniProt functional classes associated with the C-SRC and N1-SRC interacting proteins are similar (Extended Data Figs. 1-3, 1-4, Sheet 1). Figure 1*A* shows that these functional classes are represented within both cohorts in similar proportions. Figure 1*B* is a STRING network, with N1-SRC SH3 ligands highlighted within the larger C-SRC network. SRC SH3 ligands form a large functional association network, indicating that many of these proteins are involved in regulating similar cellular processes. Identified SRC SH3 ligands are associated with known functions of SRC in regulating the cytoskeleton, signaling, membrane trafficking, and gene expression. Notably, our analysis also indicates that a large group of ligands have functions associated with RNA metabolism and processing (C-SRC = 38/176; N1-SRC = 8/33). GO analyses of SRC interactors also show enrichment for multiple terms associated with RNA metabolism and processing (Extended Data Figs. 1-3, 1-4, Sheet 2).

10.1523/JNEUROSCI.1705-24.2025.f1-1 Figure 1-1 Proteomics analysis pipeline used in the study. Flow diagram summarising the approaches used to analyse the LC-MS/MS data arising from the C- and N1-SRC SH3 domain pulldown experiment. Download Figure 1-1, TIF file.

**Figure 2.**
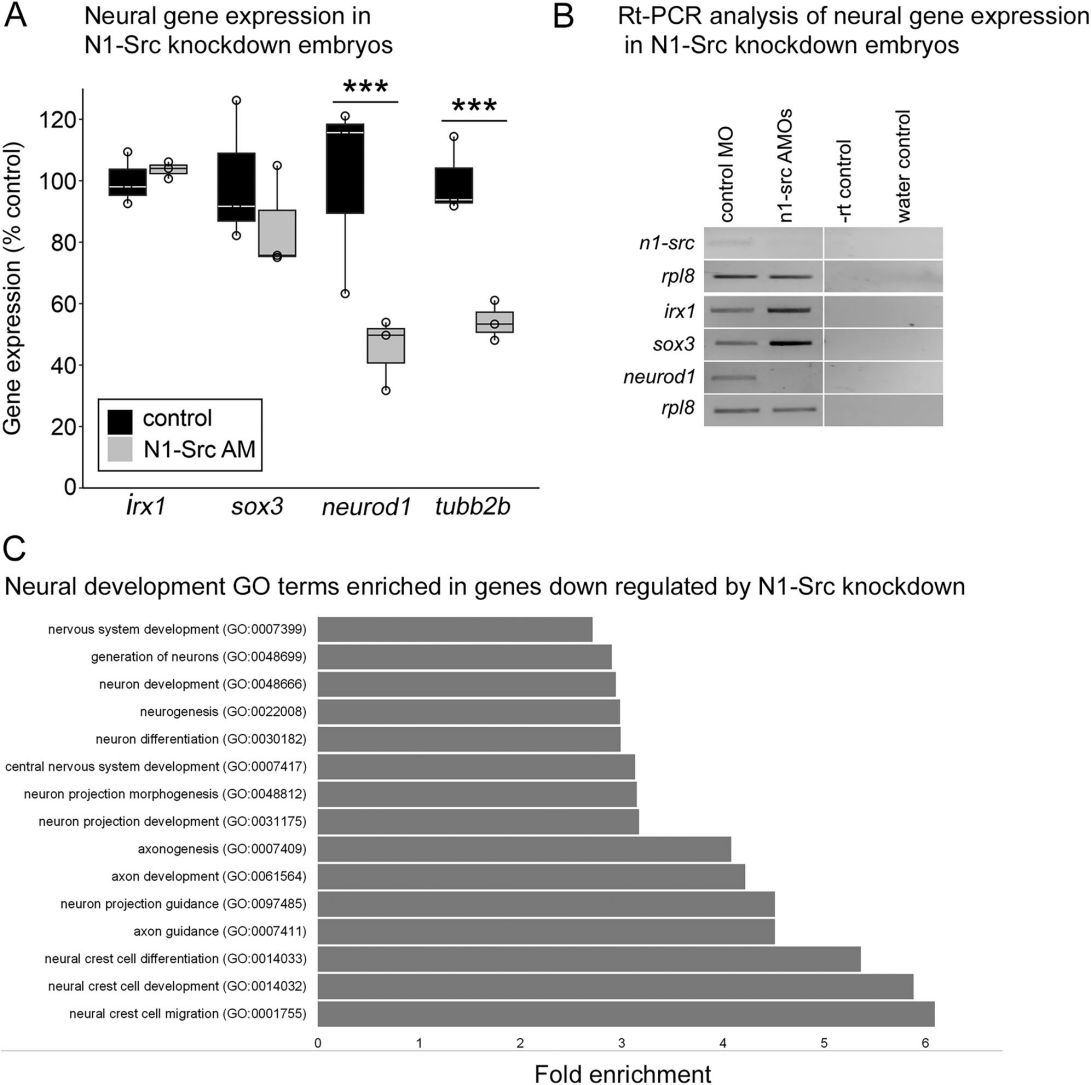
Expression levels of neural development genes are downregulated in N1-SRC morphants. ***A***, A box and whisker plot of the relative expression in control and N1-SRC morphants of neural prepattern genes *irx* and *sox3*, the proneural gene *neurod1*, and the neuronal differentiation marker gene *tubb2b* as determined by RNA-seq analysis at Midneurula Stage 16. Illumina sequencing reads were mapped and quantified using Salmon. Mean expression values in TPM calculated from Salmon gene-level output. Significance is indicated by *q* values/FDRs as calculated by Sleuth analysis of Salmon gene-level output. ***B***, RT-PCR analysis of neural gene expression in control and N1-SRC morphant embryos at Midneurula Stage 16. ***C***, Fold enrichment of neural development-related PANTHERdb Biological Process GO terms associated with genes downregulated in *n1-src* morphants (*q* ≤ 0.05; log2[effect size] < 0). (See Extended Data Fig. 2-1 for a schematic of the transcriptomics pipeline employed, Extended Data Fig. 2-2 for data relating to the effectiveness of N1-SRC knockdown in morphants, Extended Data Fig. 2-3 for an analysis of gene expression in morphants, and Extended Data Fig. 2-4 for GO analysis of genes significantly downregulated in morphants.)

10.1523/JNEUROSCI.1705-24.2025.f1-2 Figure 1-2 **Mass spectrometry data** **Sheet 1.** Analysis of LC-MS/MS data from GST (control), C-SRC-SH3 and N1-SRC-SH3 pulldown. Data include protein ID, spectral counts, statistical tests to determine significant binding partners and semi-quantitative analysis of protein abundance (emPAI). Download Figure 1-2, XLSX file.

**Figure 3.**
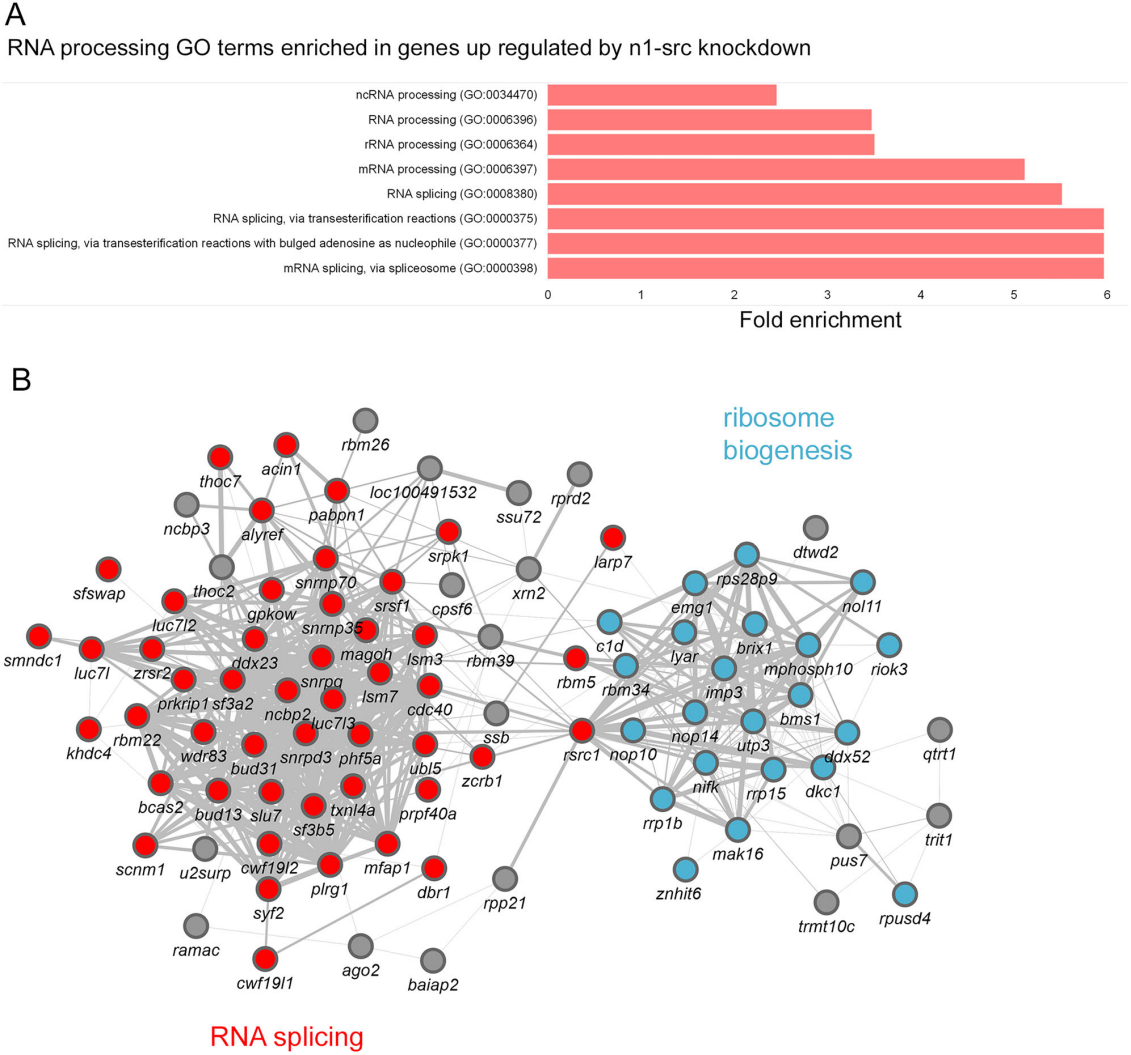
Expression levels of RNA processing genes are upregulated in N1-SRC morphants. ***A***, Fold enrichment of RNA processing PANTHERdb Biological Process GO terms associated with genes upregulated in *n1-src* morphants (*q* ≤ 0.05; log2[effect size] > 0). ***B***, Functional association network of genes belonging to RNA processing GO terms that are upregulated in N1-SRC morphants. Edge thickness is proportional to physical interaction data from STRING-db. Nodes are colored according to the function: red, RNA splicing; blue, ribosome biogenesis; gray, other. (See Extended Data Fig. 3-1 for GO analysis of genes significantly upregulated in morphants.)

10.1523/JNEUROSCI.1705-24.2025.f1-3 Figure 1-3 **Functional analysis of C-SRC SH3 domain interacting proteins** **Sheet 1.** Annotation of C-SRC SH3 interacting proteins using PANTHER, UniProt, STRING and the Rat genome databases. **Sheet 2.** PANTHER Biological Process Gene Ontology term analysis of C-SRC interacting proteins. Download Figure 1-3, XLSX file.

**Figure 4.**
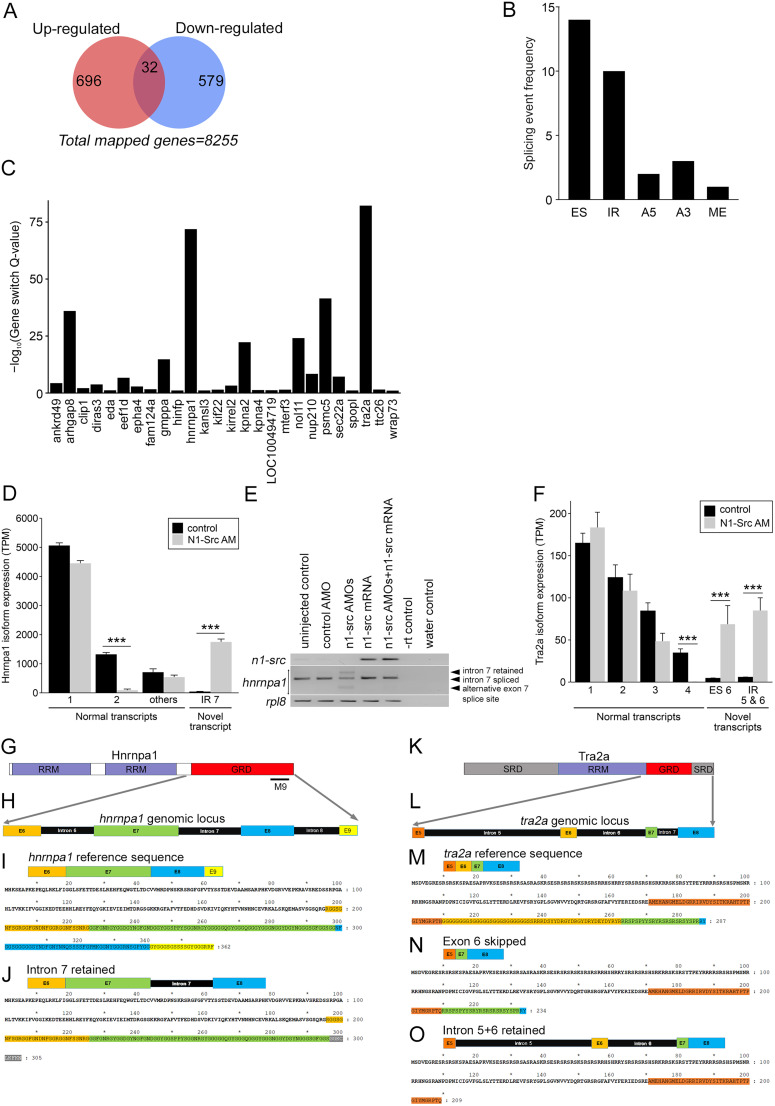
Transcript isoform usage is altered in a subset of genes in N1-SRC morphant embryos. ***A***, A Venn diagram showing the overlap of genes with both up- and downregulated transcripts in N1-SRC morphants. ***B***, Frequency histogram of splicing events altered in *n1-src* morphants. ES, exon skipping; IR, intron retention; A5, altered 5′ splice site; A3, altered 3′ splice sites; ME, mutually exclusive exon splicing. ***C***, A plot of the Gene Switch *Q*-value calculated by IsoformSwitchAnalyzeR for each validated isoform switch in *n1-src* morphants. ***D***, The relative abundance of *hnrnpa1* transcripts from IsoformSwitchAnalyzeR plotted for controls and *n1-src* morphants. Novel transcripts in *n1-src* morphants are grouped together. Statistically significant comparisons of differential expression between control and *n1-src* are indicated; ***p < 0.001. Standard error bars are shown. ***E***, RT-PCR analysis of *n1-src*, *hnrnpa1*, and loading control *rpl8* transcripts at Neurula Stage 16 in uninjected control, control morpholino injected, *n1-src* splice-blocking morpholinos injected, *n1-src* mRNA injected, and *n1-src* splice-blocking morpholinos and *n1-src* mRNA coinjected embryos. Black arrows indicate different *hnrnpa1* splice isoforms. ***F***, The relative abundance of *tra2a* transcripts from IsoformSwitchAnalyzeR plotted for controls and *n1-src* morphants. ***G***, Domain structure of the Hnrnpa1 protein: GRD, glycine-rich domain; RRM, RNA recognition motif. M9, nuclear import/export regulatory sequence. ***H***, Detail of the exon/intron structure of the carboxy terminal, the GRD encoding region of *hnrnpa1*. ***I***, The Hnrnpa1 reference protein sequence. Regions encoded by exons 6, 7, and 8 are indicated. ***J***, The conceptual peptide sequence encoded by *hnrnpa1* transcripts with retained intron 7. ***K***, Domain structure of the Tra2a protein: GD, glycine-rich domain; RRM, RNA recognition motif; SRD, serine-/arginine-rich domain. ***L***, Detail of the exon/intron structure of the carboxy terminal, glycine-rich, and serine-rich encoding region of *tra2a*. ***M***, The Tra2a reference protein sequence. Regions encoded by exons 5, 6, 7, and 8 are indicated. ***N***, The conceptual peptide sequence encoded by *tra2a* transcripts with skipped exon 6. ***O***, The conceptual peptide sequence encoded by *tra2a* transcripts with retained intron 5 and 6. (See Extended Data Fig. 4-1 for the LR transcriptome, Extended Data Fig. 4-2 for an analysis of transcript expression in morphants, Extended Data 4-3 for additional data relating to genes with altered splicing in morphants, Extended Data Fig. 4-4 for GO analysis of transcripts differentially expressed in morphant embryos, and Extended Data Fig. 4-5 for data relating to the IsoformSwitchAnalyzeR-based analysis of altered splicing events.)

10.1523/JNEUROSCI.1705-24.2025.f1-4 Figure 1-4 **Functional analysis of N1-SRC SH3 domain interacting proteins** **Sheet 1.** Annotation of N1-SRC SH3 interacting proteins using PANTHER, UniProt, STRING and the Rat genome databases. **Sheet 2.** PANTHER Biological Process Gene Ontology term analysis of N1-SRC interacting proteins. Download Figure 1-4, XLSX file.

A role in RNA processing represents a novel, unexplored function of SRC proteins. The subset of C-SRC SH3 ligands with RNA processing/metabolism functions forms a highly connected hub within the larger C-SRC association network (Fig. 1*C*). Similarly, the network of N1-SRC SH3 domain interactors contains a hub of RNA metabolism and processing proteins (Fig. 1*D*), including the paraspeckle proteins NONO and SFPQ; splicing factors SF1, SF3B2, and WBP11; and members of the cleavage factor Im complex, CPSF6, CPSF7, and NUDT21, associated with alternative polyadenylation.

For a subset of the SH3 ligands, the DNM1 trafficking protein, the WASF1 cytoskeletal regulator, and the SF1 splicing factor, we validated the mass spectrometry data by Western blotting of the lysate pulldown eluates (Fig. 1*E*). While all three proteins associate with both SRC SH3 domains, there is a reduced association with N1-SRC SH3, suggesting that these ligands bind N1-SRC SH3 with lower affinity than C-SRC SH3. Calculating the relative abundance of C-SRC and N1-SRC SH3 binding partners by semiquantitative analysis of the tryptic peptides recovered in the pulldown assay also indicates that the majority of interactors have a lower affinity for N1-SRC SH3 than C-SRC SH3, with just a few targets binding to a similar extent (Fig. 1*F*; Extended Data Fig. 1-2). These findings suggest the microexon insert in the N1-SRC SH3 domain does not confer specificity for novel SH3 binding but broadly reduces C-SRC SH3 domain ligand binding affinity and there are no unique N1-SRC SH3 domain interactors.

### Effective knockdown of *n1-src* transcripts in N1-SRC morphants

*n1-src* is expressed in the neural plate of the *Xenopus* embryo, and we have previously shown that it is required for the differentiation of neurons in the primary nervous system (Lewis et al., 2017). Injection of antisense morpholino oligos targeting the splice acceptor and donor sites of the *Xenopus n1-src* microexon into developing embryos knocks down *n1-src* expression, but does not affect that of *c-Src* (Lewis et al., 2017).

Based on our observations, we hypothesized that N1-SRC has a role in regulating RNA processing and metabolism during development of the nervous system. We therefore analyzed changes in the transcriptome in morpholino oligo-mediated N1-SRC knockdown *Xenopus* embryos (morphants) during neural development. Illumina SR RNA-seq–based expression analysis was carried out on N1-SRC morphant embryos during neuronal differentiation at Midneurula Stage 16 (Extended Data Fig. 2-1). Prior to RNA-seq analysis, inhibition of *n1-src* expression in morphant embryo batches was confirmed by RT-PCR (Extended Data Fig. 2-2*A*). Differential gene expression analysis of the triplicate RNA-seq dataset shows a significant reduction in overall *Src* gene expression (*q* < 0.01; b(log2[effect size]) = −0.27; Extended Data Fig. 2-3). Furthermore, mapping of SR sequences to the region of the *Xenopus src* locus containing the *n1-src* microexon shows a peak in control embryos, which is absent in morphant embryos (Extended Data Fig. 2-2*B*), confirming that expression of *n1-src* is reduced in morphants.

### Downregulation of neurodevelopmental genes in N1-SRC morphants

We previously observed by in situ hybridization that expression of the neuronal differentiation marker *tubb2b* is downregulated in N1-SRC morphants (Lewis et al., 2017). Gene-level expression analysis of the SR RNA-seq data (Extended Data Fig. 2-3) confirms that *tubb2b* expression is reduced in morphants (Fig. 2*A*). Furthermore, we show that the proneural gene *neurod1* is significantly downregulated in morphants, whereas expression of neural prepattern genes *sox3* and *irx1* does not change significantly (Fig. 2*A*). These observations were confirmed by independent RT-PCR analysis (Fig. 2*B*). Downregulation of *neurod1* and *tubb2b*, but not *sox3* or *irx1*, indicates that *Xenopus* N1-SRC is not required for the establishment of the neural territory but is required for subsequent neural fate specification and differentiation. In keeping with the requirement for N1-SRC in the development of the nervous system, PANTHERdb GO analysis shows enrichment of neural developmental terms associated with genes significantly downregulated in morphants (*q* ≤ 0.05; *b* < 0). For example, the neuron differentiation term (GO:0030182) and neurogenesis term (GO:0022008) are enriched ∼3-fold, with FDRs <1.0 × 10^−8^ (Fig. 2*C*; Extended Data Fig. 2-4, gray highlight).

10.1523/JNEUROSCI.1705-24.2025.f2-1 Figure 2-1 **Transcriptomic analysis pipeline used in the study** Flow diagram showing the multiple approaches used in this study to determine differential gene, transcript and splice variant expression in control and N1-SRC morphant embryos. Download Figure 2-1, TIF file.

10.1523/JNEUROSCI.1705-24.2025.f2-2 Figure 2-2 Injection of *n1-src* splice blocking antisense morpholinos effectively blocks N1-Src expression. **A-** rt-PCR analysis of *n1-src* expression in the three sets of neurula stage 16 control morpholino (control MO) injected and n1-src antisense, splice blocking morpholino (n1-src AMO) injected embryos used for RNA-Seq analysis. *Rpl8* is used as a loading control. **B-** Mapping of Illumina short read sequences to n1-src microexon in control MO and n1-src AMO embryos. Download Figure 2-2, TIF file.

10.1523/JNEUROSCI.1705-24.2025.f2-3 Figure 2-3 **Analysis of differential gene expression in control versus N1-SRC morphant embryos** Illumina reads were aligned to the *Xenopus tropicalis* reference transcriptome with Salmon. Transcript counts were aggregated to estimate gene expression levels. Differential gene expression in control versus n1-src morphant embryos was analysed by Sleuth. **Sheet 1.** Full unfiltered dataset. **Sheet 2.** Genes with mean expression values >=1.0 TPM in either control or n1-src morphant embryos. **Sheet 3.** Genes with significant differential expression between control and n1-src morphant embryos, False discovery rate (q) <=0.05. **Sheet 4.** Genes significantly up regulated in n1-src morphant versus control embryos, b ([ Log2[effect size]) > 0, q<=0.05) **Sheet 5.** Genes significantly down regulated in n1-src morphant versus control embryos, b ([ Log2[effect size]) < 0, q<=0.05) Download Figure 2-3, XLSX file.

10.1523/JNEUROSCI.1705-24.2025.f2-4 Figure 2-4 **Gene ontology analysis of genes significantly down regulated in N1-SRC morphant versus control embryos** **Sheet 1.** Output of PANTHERdb analysis of biological process Gene Ontology (GO) terms for significantly down regulated genes (Extended Data Figure 2-3, Sheet 5). **Sheet 2.** Additional stringent filtering was undertaken to highlight the most significantly enriched biological process GO terms in down regulated genes (input gene number >=10, enrichment >=2, false discovery rate<=0.01). Grey shading indicates terms associated with neural development. Download Figure 2-4, XLSX file.

### Upregulation of RNA processing genes in N1-SRC morphants

Interestingly, given the hypothesis that N1-SRC has a function related to RNA processing, GO term analysis of genes upregulated (*q* ≤ 0.05; *b* > 0) in morphants indicates enrichment of terms associated with mRNA splicing and processing (Fig. 3*A*; Extended Data Fig. 3-1, red highlight). Thus, the mRNA splicing term (GO:0000398) and mRNA processing term (GO:0006397) are enriched by >5-fold and FDRs < 5.0 × 10^−14^. This suggests that not only does N1-SRC associate with RNA processing proteins; it is also involved in regulating the expression of RNA processing genes. The genes upregulated by N1-SRC knockdown and associated with RNA processing GO terms in Figure 3*A* form a highly connected functional association network with two main hubs; a larger one comprising RNA splicing genes and a smaller one of genes involved in ribosome biogenesis (Fig. 3*B*).

10.1523/JNEUROSCI.1705-24.2025.f3-1 Figure 3-1 **Gene ontology analysis of genes significantly up regulated in N1-SRC morphant versus control embryos** **Sheet 1.** Output of PANTHERdb analysis of biological process Gene Ontology (GO) terms for significantly up regulated genes (Extended Data Figure 2-3, Sheet 4). **Sheet 2.** Additional stringent filtering was undertaken to highlight the most significantly enriched biological process GO terms in up regulated genes (input gene number >=10, enrichment >=2, false discovery rate<=0.01). Red shading indicates terms associated with RNA processing. Download Figure 3-1, XLSX file.

### mRNA splicing is altered in N1-SRC morphants

Given the proposed role of N1-SRC in regulating RNA processing, we investigated whether mRNA splicing is altered in N1-SRC morphants. We reasoned it is likely that altered splicing in morphants includes novel events not represented in the reference *Xenopus* transcriptome. In order to characterize transcripts containing such novel splice events, a new transcriptome was assembled based on Nanopore LR sequencing of mRNA extracted from control and N1-SRC morphants. The rationale for creating a LR transcriptome is that it provides a view of splice junction combinations present in individual transcripts, which is not possible with a transcriptome based on SR sequence reads of ∼150 bp. The assembled LR-based transcriptome identified 14,729 transcripts from 8,255 uniquely mapped annotated genes (Extended Data Fig. 4-1).

Despite the advantages of the LR transcriptome for identifying the sequence of actual transcripts present in embryos, the relatively low sequencing depth means that it does not allow for effective transcript quantification. To overcome this limitation, quantification and differential transcript expression analysis was undertaken by mapping the original Illumina SR sequencing to the new LR transcriptome (Extended Data Fig. 4-2). This approach is discriminating and in keeping with our previous study (Lewis et al., 2017) detects a 6.6-fold reduction in *n1-src* (*src.3*) transcript expression in morphant embryos; in contrast, *c-src* transcripts (*src.1* and *src.2*) are less affected (1.1- and 1.4-fold reductions, respectively; Extended Data Figs. 4-3*A*,*B*).

The analysis revealed 632 transcripts, from 611 genes, are significantly downregulated and 775 transcripts, from 728 genes, are significantly upregulated (Extended Data Fig. 4-2). As expected, GO term analysis of the 1,307 genes with transcripts significantly changing (up and down) in N1-SRC morphants shows enrichment for terms associated with both mRNA processing (red shading) and neural development (gray shading; Extended Data Fig. 4-4). Some genes have both significantly up- and downregulated transcripts in morphants, which suggests that mRNA splicing is altered by N1-SRC knockdown. Figure 4*A* shows that of the 1,307 genes with transcripts significantly changing in morphants, 32 have both up- and downregulated transcripts, including genes for the splicing factors HNRNPA1 and TRA2A (Extended Data Fig. 4-3*C*).

10.1523/JNEUROSCI.1705-24.2025.f4-1 Figure 4-1 **Nanopore long read RNA-seq based transcriptome** A Nanopore long read transcriptome was assembled using HISAT2 and StringTie. Assembled transcripts were merged back to the reference annotation with gffcompare to link gene names to the assembled StringTie transcripts. The sequence of all StringTie transcripts mapping to known genes are shown. Download Figure 4-1, XLSX file.

10.1523/JNEUROSCI.1705-24.2025.f4-2 Figure 4-2 **Analysis of differential transcript expression in control versus N1-SRC morphant embryos** Illumina reads were aligned to the novel Nanopore long read *Xenopus tropicalis* transcriptome (Extended Data Figure 4-1) with Salmon. Differential transcript expression in control versus n1-src morphant embryos was analysed by Sleuth. **Sheet 1.** Transcripts mapping to known genes **Sheet 2.** Transcripts with mean expression values >=1.0 TPM in either control or n1-src morphant embryos. **Sheet 3.** Transcripts with significant differential expression between control and n1-src morphant embryos, False discovery rate (q) <=0.05. **Sheet 4.** Transcripts significantly up regulated in n1-src morphant versus control embryos, b ([ Log2[effect size]) > 0, q<=0.05) **Sheet 5.** Transcripts significantly down regulated in n1-src morphant versus control embryos, b ([ Log2[effect size]) < 0, q<=0.05) Download Figure 4-2, XLSX file.

10.1523/JNEUROSCI.1705-24.2025.f4-3 Figure 4-3 **Altered splicing in N1-SRC knockdown embryos** **A-** bar chart showing the relative expression of Src splice variants (*c-src.1, c-src.2* and *n1-src*) in control and N1-SRC morphant embryos. Illumina sequencing reads were mapped to the Nanopore long read transcriptome and quantified using Salmon. Mean expression values in transcripts per million (TPM) calculated from Salmon transcriptome level output are included. **B-** conceptual peptide sequences of C-SRC.1, C-SRC.2 and N1-SRC splice variants. **C-** a list of genes which have transcripts both up and down regulated in n1-src knockdown embryos. Genes with functions associated with RNA metabolism and processing are shaded red. **D-** *in situ* hybridization analysis of *tra2a* expression during *Xenopus* development. (*i*) is a dorsal view of the neural plate of a neurula stage 16 embryo. (*ii)* is a lateral view (anterior to the left) of an early tailbud stage embryo, *bra* = branchial arch, *hdb* = hindbrain. **E-** Cumulative Nanopore long read sequences from control and n1-src knockdown embryos mapped to the region of retained intron 7 of *hnrnpa1*. **F-** Cumulative Nanopore long read sequences from control and n1-src knockdown embryos mapped to the region of skipped exon 6 and retained introns 5 and 6 of *tra2a*. Download Figure 4-3, TIF file.

10.1523/JNEUROSCI.1705-24.2025.f4-4 Figure 4-4 **Gene ontology analysis of transcripts differentially expressed in N1-SRC morphant versus control embryos** **Sheet 1.** Output of PANTHERdb analysis of biological process Gene Ontology (GO) terms for significantly up regulated genes (Extended Data Figure 4-2, Sheet 4). **Sheet 2.** Additional stringent filtering was undertaken to highlight the most significantly enriched biological process GO terms in up regulated genes (input gene number >=10, enrichment >=2, false discovery rate<=0.01). Grey shading indicates terms associated with neural development. Red shading indicates terms associated with RNA processing. Download Figure 4-4, XLSX file.

**Figure 5.**
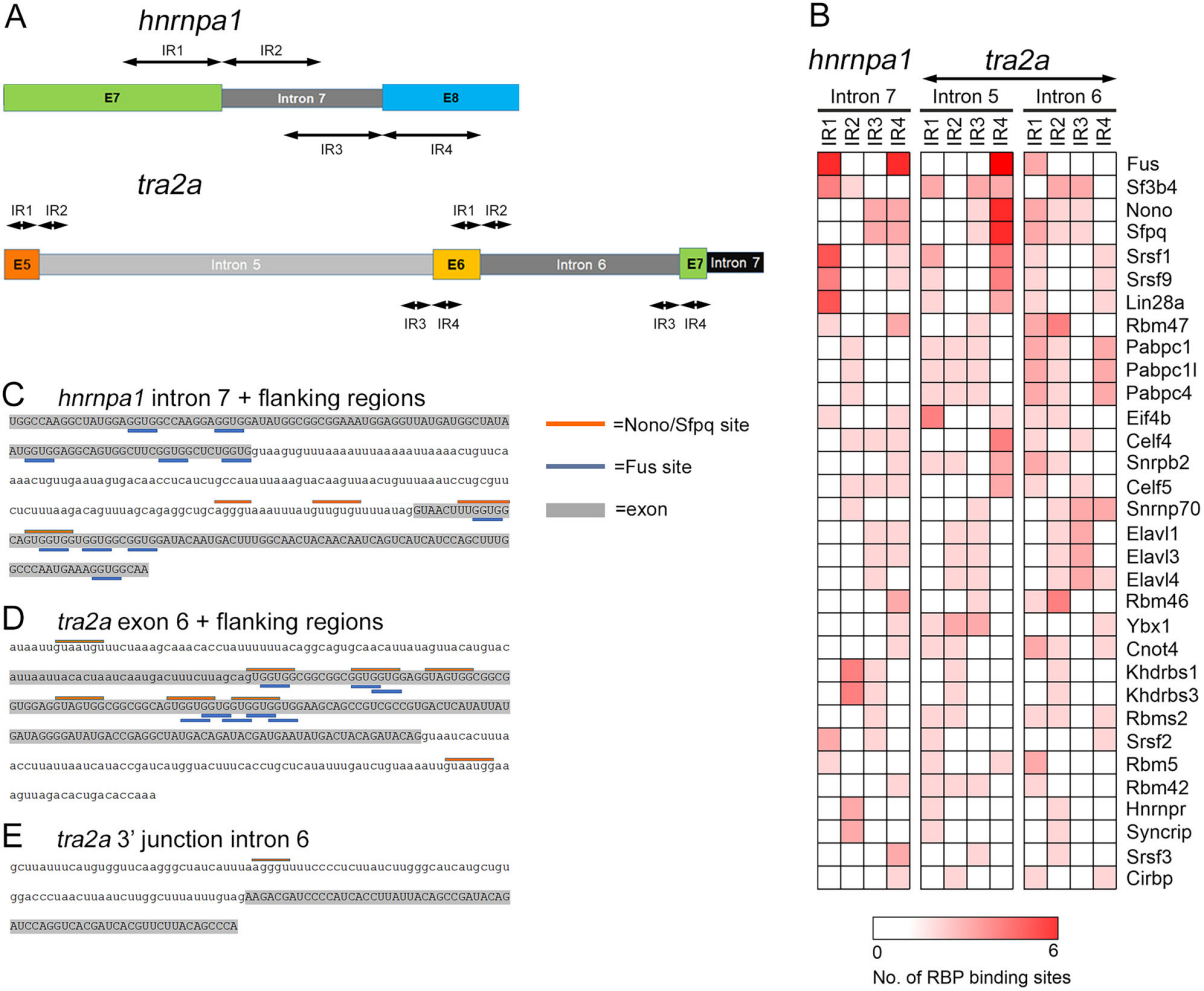
RBPs identified as SRC kinase substrates have consensus binding sites at splice junctions in Hnrnpa1 and Tra2a. ***A***, The regions with altered splicing of the *hnrnpa1* and *tra2a* transcripts. The 100 base regions flanking the 5′ end of affected introns are designated IR1 and IR2. The 100 base regions flanking the 3′ end of affected introns are designated IR3 and IR4. ***B***, Heatmap of consensus RBP sites identified in the IR1-IR4 regions of *hnrnpa1* intron 7 and *tra2a* introns 5 and 6 (scale bar, 0–6 site counts). Singleton RBP sites were eliminated from the analysis (only RBP sites present in >1 region are included). ***C***, Consensus Nono/Sfpq heterodimer and Fus binding sites in the intron 7 region of *hnrnpa1*. Exon sequence is shaded gray and in uppercase. ***D***, Consensus Nono/Sfpq heterodimer and Fus binding sites in the skipped exon 6 region of *tra2a*. ***E***, Consensus Nono/Sfpq heterodimer and Fus binding sites in the retained intron 6 region of *tra2a*. (See Extended Data Figs. 5-1 and 5-2 for data relating to the analysis of *hnrnpa1* and *tra2a* splice junctions for consensus RBP sites.)

10.1523/JNEUROSCI.1705-24.2025.f4-5 Figure 4-5 **Splice event analysis** **Sheet 1.** IsoformSwitchAnalyzeR output, identifying significant isoform switch events between control and n1-src morphant embryos. Splice event types were confirmed manually and genomic coordinates were obtained using Astalavista. Download Figure 4-5, XLSX file.

### Exon skipping and intron retention is increased in N1-SRC morphants

Given our observations, a global analysis of the effects of N1-SRC knockdown on mRNA splicing was undertaken. Our pipeline used alignments of the Illumina SR sequences to the Nanopore LR transcriptome to identify transcripts with splice junctions altered in N1-SRC morphants (Extended Data Fig. 2-1). A manual inspection of flagged changes generated a list of 26 genes with well-supported altered splicing in morphants (Extended Data Fig. 4-5). The most commonly detected changes were exon skipping and intron retention (Fig. 4*B*). Figure 4*C* shows the calculated significance (*q* values) of isoform switching events for genes with changed transcript isoform usage.

### Altered *hnrnpa1* and *tra2a* splicing in N1-SRC morphants

Prominent among the genes with altered splicing in N1-SRC morphants are *hnrnpa1* and *tra2a*, which exhibit the most highly significant changes in isoform usage (Fig. 4*C*). *hnrnpa1* encodes an RBP with expression enriched in the developing *Xenopu*s neural plate and neural tube (Dichmann et al., 2008). The graph in Figure 4*D* shows an analysis of changes in the proportions of *hnrnpa1* transcripts present in control and morphant embryos. Of particular note is a highly significant increase in morphants of a novel transcript containing a retained intron 7. This is graphically represented in Extended Data Figure 4-3*E*, which shows increased mapping of Nanopore LR sequences to intron 7 of *hnrnpa1*. RT-PCR and amplicon sequencing shows that intron 7 retention is very low in control embryos, but this increases in N1-SRC morphants (Fig. 4*E*). Another smaller, mis-spliced product is detected, which corresponds to transcripts spliced using an alternative 5′ splice site in exon 7. To test the specificity of the observed effects on splicing, *Xenopus n1-src* mRNA was coinjected with the splice-blocking morpholinos. Coinjection of *n1-src* mRNA rescues both effects on *hnrnpa1* splicing resulting from N1-SRC knockdown (Fig. 4*E*).

In situ hybridization analysis shows that, like *hnrnpa1*, *Xenopus tra2a* is expressed in the neural plate at the neurula stage and neural expression continues in tailbud stage embryos (Extended Data Fig. 4-3*Di*–*iii*). *Tra2a* is also expressed at high levels in the branchial arches in tailbud embryos (Extended Data Fig. 4-3*Dii*). Figure 4*F* indicates that there is increased skipping of *tra2a* exon 6 and retention of introns 5 and 6 in morphants, and this is again reflected in increased cumulative read coverage of LR sequences in intron 5 and 6 and reduced coverage in exon 6 of the *tra2a* locus in morphant versus control embryos (Extended Data Fig. 4-3*F*).

The consequences of the observed mis-splicing events on the encoded HNRNPA1 and TRA2A proteins were examined. Figure 4*G*–*J* shows that *hnrnpa1* intron 7 retention leads to a frame shift and a truncated protein lacking a C-terminal glycine–rich domain (GRD) present in the reference sequence. Figure 4*K*–*N* shows that exon 6 of *tra2a* has the properties of a cassette exon. Skipping of exon 6 generates a transcript encoding a conceptual in-frame TRA2A protein lacking a 53 amino acid GRD found in the reference sequence. Transcripts with retained intron 5 and 6 encode a protein that is truncated before the GRD (Fig. 4*O*).

### Putative sites for SFPQ/NONO and FUS are present at splice junctions affected by N1-SRC knockdown

A possible mechanism for N1-SRC regulation of splicing is that it phosphorylates and alters the activity of splicing factors bound to the regions of affected splice sites. To investigate this possibility, we interrogated 100 base regions of sequence around the affected splice junctions for the presence of consensus RBP sites [Extended Data Figs. 5-1 (*hnrnpa1*), 5-2 (*tra2a*)]. Figure 5*A* shows the strategy used for analyzing the splice junctions at the 5′ end (IR1+IR2) and the 3′ end (IR3 + IR4) of *hnrnpa1* intron 7 and *tra2a* introns 5 and 6, including 5′ and 3′ splice junctions of exon 6.

10.1523/JNEUROSCI.1705-24.2025.f5-1 Figure 5-1 RNA binding protein (RBP) site analysis of *hnrnpa1* splice junctions affected in N1-SRC morphant embryos **Sheet 1.** CISBP-RNA database analysis of IR1-4 of *hnrnpa1* intron 7 **Sheet 2.** RBP site analysis of indicated sequence from *hnrnpa1* intron 7 and its flanking regions. Download Figure 5-1, XLSX file.

10.1523/JNEUROSCI.1705-24.2025.f5-2 Figure 5-2 RNA binding protein (RBP) site analysis of *tra2a* splice junctions affected in N1-SRC morphant embryos **Sheet 1.** CISBP-RNA database analysis of IR1-4 of tra2a introns 5 and 6. **Sheet 2.** RBP site analysis of indicated sequence from 5’ end of intron 5 and its flanking regions. **Sheet 3.** RBP site analysis of indicated sequence from exon 6 and its flanking regions. **Sheet 4.** RBP site analysis of indicated sequence from 3’ end of intron 6 and its flanking regions. Download Figure 5-2, XLSX file.

Figure 5*B* is a heatmap of consensus RBP sites identified using the CISBP-RNA database. The most abundant consensus binding sites at the affected junctions are those for FUS, which has previously been shown to be a target of ABL and SRC family kinase phosphorylation (Darovic et al., 2015; Motaln et al., 2023). Multiple binding sites for the SFPQ/NONO heterodimer were also identified in both *hnrnpa1* and *tra2*. Interestingly, both SFPQ and NONO are N1-SRC SH3 domain ligands identified in our interactor screen (Fig. 1). Figure 5*C*–*E* shows the position of FUS and SFPQ/NONO sites in the regions of *Xenopus hnrnpa1* intron 7 and intron 5/exon 6/intron 6 of *tra2a*.

### Components of the splicing machinery are phosphorylated by SRC kinases

**Figure 6.**
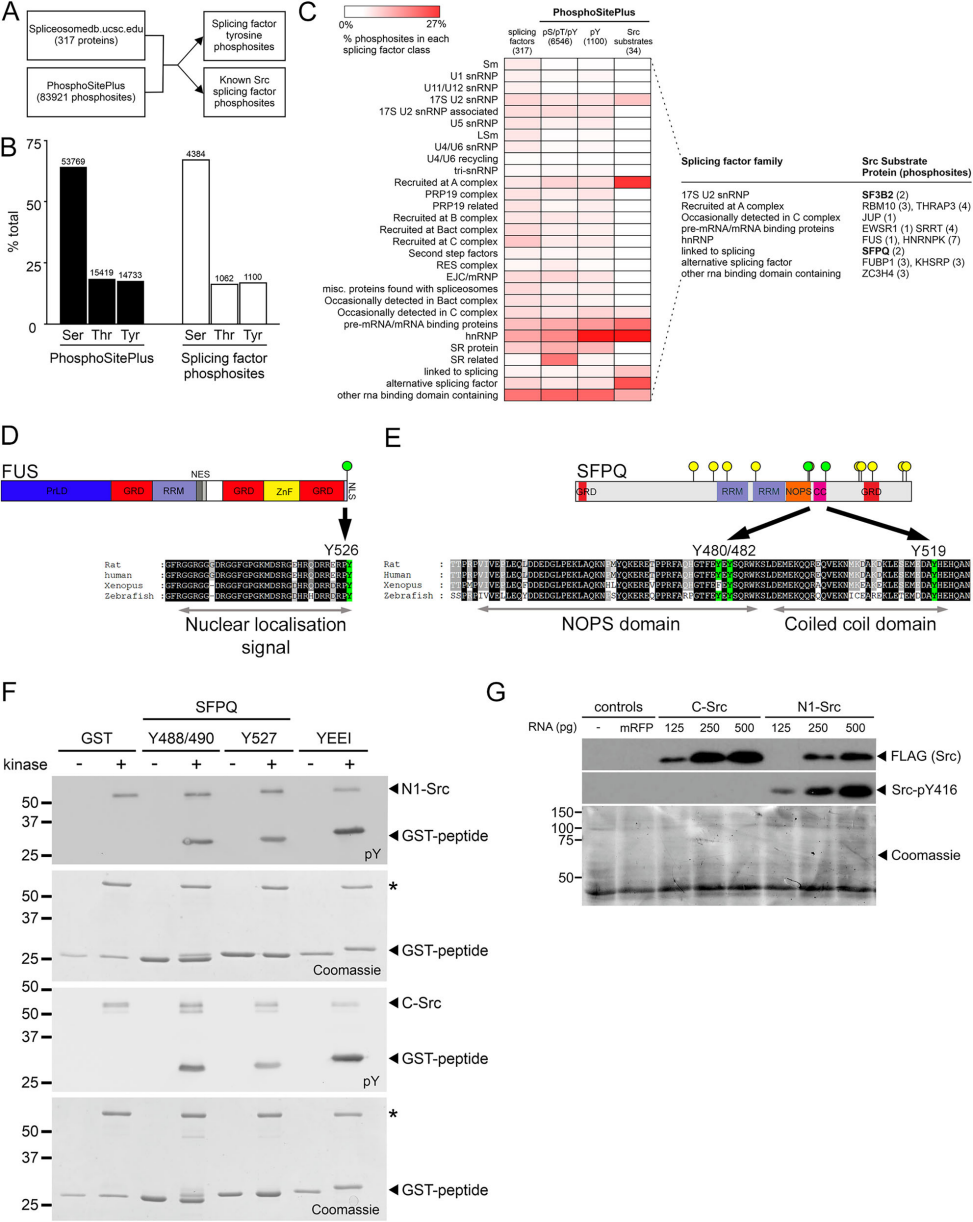
The splicing machinery is phosphorylated by SRC kinases. ***A***, The pipeline for the bioinformatic analysis to determine SRC-dependent substrates in the splicing machinery in the PhosphoSitePlus database. ***B***, A plot of the percentage of serine, threonine, and tyrosine phosphosites in the complete PhosphoSitePlus database (left bars) or for proteins identified as splicing machinery (right bars). ***C***, Heatmap depicting the percentage of proteins (Column 1 only) or tyrosine phosphosites attributed to different subclasses of the splicing machinery within the indicated datasets. The number of proteins analyzed in each dataset is indicated. The table (right) summarizes the splicing factors that have been experimentally determined to be Src substrates. ***D***, Domain structure of the FUS protein with a multiple sequence alignment of FUS nuclear localization signal sequences from rat, human, *Xenopus*, and zebrafish showing conserved C-terminal phosphotyrosine residue. GRD, glycine-rich domain; NES, nuclear export signal; NLS, nuclear localization signal; PrLD, prion-like domain; RRM, RNA recognition motif; ZnF, zinc finger domain. ***E***, Domain structure of the SFPQ protein with a multiple alignment showing conservation of peptide sequences in the NOPS and coiled domain in rat, human, *Xenopus*, and zebrafish SFPQ. Green coloring indicates conserved phosphotyrosine sites. Yellow lollipops indicate phosphotyrosine residues identified in the PhosphoSitePlus database and green are predicted Src sites. GRD, glycine-rich domain; NOPS, NONA/ParaSpeckle domain; RRM, RNA recognition motif. ***F***, In vitro C-SRC and N1-SRC kinase assays using GST-peptide fusions of SFPQ, encoding predicted Src consensus motifs. Phosphorylation was detected by immunoblotting with a phosphotyrosine antibody (pY), and recombinant protein levels in each assay were detected by Coomassie staining. GST was included as a negative control, and GST-YEEI is an ideal Src substrate. The asterisk indicates autophosphorylation of SRC proteins. ***G***, Western blot of Neurula Stage 17 *Xenopus* embryos injected with the indicated amounts of synthetic C-SRC and N1-SRC mRNA. Uninjected embryos and embryos injected with 500 pg monomeric red fluorescent protein (mRFP) are included as negative controls. Translated SRC proteins are detected by the FLAG epitope tag. Activity of SRC proteins is detected by autophosphorylation of Y416. Coomassie staining of total loaded proteins is used as a loading control. (See Extended Data Fig. 6-1 for data relating to the phosphosite analysis of RNA splicing proteins and Extended Data Fig. 6-2 for the phenotypes of *Xenopus* embryos injected with *C-Src* and *N1-Src* mRNA.)

To assess whether N1-SRC tyrosine phosphorylation of RBPs might be a widespread mechanism for regulating splicing, we analyzed publicly available phosphoproteomics data. The pipeline employed is outlined in Figure 6*A*. The 1,100 phosphotyrosine sites were identified in splicing machinery proteins (Fig. 6*B*). Thirty-six SRC phosphosites were identified in 14 splicing-related proteins, of which, 30 are conserved in the corresponding *Xenopus* proteins (Extended Data Fig. 6-1). Figure 6*C* is a heatmap of the data from these phosphosite analyses, classified by splicing function. There is an enrichment of tyrosine phosphosites and SRC phosphosites in hnRNPs and other RBPs, suggesting that SRC kinases act directly on splicing regulators rather than the core machinery.

10.1523/JNEUROSCI.1705-24.2025.f6-1 Figure 6-1 **Phosphosite analysis** **Sheet 1.** PhosphoSitePlus database entries for all phosphotyrosine sites identified on splicing proteins (as defined in spliceosome.db). **Sheet 2.** PhosphoSitePlus database entries for known Src substrate phosphotyrosine sites identified on splicing proteins. **Sheet 3.** Relative abundance of tyrosine phosphosites within each class of splicing factor for each PhosphoSitePlus dataset. Download Figure 6-1, XLSX file.

10.1523/JNEUROSCI.1705-24.2025.f6-2 Figure 6-2 SRC and N1-SRC overexpression phenotypes in *Xenopus* embryos. Embryos were injected at the 2-cell stage and cultured to larval stage 41. N values and percentage of abnormal embryos are indicated in each panel. **A-** normal control uninjected embryo at larval stage 41. **B-** normal embryo injected with 500 pg synthetic monomeric red fluorescent protein mRNA (mRFP). **C-** representative embryos injected with 125 pg, 250 pg and 500 pg of synthetic *C-SRC* mRNA. Most embryos are normal but mild coloboma of the iris is present in the high dose. embryo presented. **D-** representative embryos injected with 125 pg, 250 pg and 500 pg of synthetic *N1-SRC* mRNA. The majority of embryos injected with >125 pg *N1-SRC* mRNA exhibit reduced development of the eyes and abnormal development of the main body, involving axial shortening and kinking. Download Figure 6-2, TIF file.

Prominent among the identified SRC family substrates are FUS and SFPQ. Although FUS was not identified in our SH3 domain interactor screen, it is a known target of SRC phosphorylation (Motaln et al., 2023). The C-terminal SRC phosphosite (Y526) within the nuclear localization signal is highly conserved in vertebrates, including *Xenopus*. As previously shown, SFPQ and its heterodimeric binding partner NONO are C-SRC and N1-SRC interactors (Fig. 1*B*,*D*,*E*). SFPQ was previously shown to be phosphorylated by C-SRC at Y589 and Y683 (Amanchy et al., 2008), and our analysis of other tyrosines strongly phosphorylated in PhosphoSitePlus revealed Y488/490 and Y527 in the NOPS and downstream coiled coil domains, respectively, as having Src consensus motifs. Figure 6*E* shows the domain structure of SFPQ, highlighting these conserved tyrosine phosphosites. We confirmed these sites as C-SRC and N1-SRC substrates by in vitro phosphorylation of peptides containing these residues (Fig. 6*F*). NB residue numbers in Figure 6, *E* and *F*, are based on the respective rat and human sequences. We conclude that FUS and SFPQ are candidate targets of SRC phosphorylation involved in the regulation of splicing.

### N1-SRC is constitutively more active than C-SRC in embryonic cells

Our data indicate that N1-SRC binds to a subset of C-SRC ligands, and in vitro, both kinases are able to phosphorylate a target protein such as SFPQ. However, neurogenesis is inhibited in N1-SRC knockdown *Xenopus* embryos despite C-SRC expression being largely unaffected (Lewis et al., 2017). Thus, C-SRC is unable to substitute for N1-SRC during *Xenopus* development, which raises further questions regarding the mechanism underpinning N1-SRC–specific functions.

We previously reported that N1-SRC has higher kinase activity than C-SRC in rat neuroblastoma cells (Keenan et al., 2015). Here we investigated whether this is also the case in cells of the developing *Xenopus* embryo. Autophosphorylation of SRC-Y416 is an important step in activating the kinase domain and is a proxy for SRC kinase activity. Figure 6*G* shows that Y416 phosphorylation is not detectable in embryos injected with up to 500 pg of C-SRC mRNA. However, injection of increasing amounts of *n1-src* mRNA leads to a dramatic increase in Y416 phosphorylation, indicating that N1-SRC has a much higher kinase activity than C-SRC. This is also reflected in the phenotypes of embryos resulting from SRC overexpression (Extended Data Fig. 6-2). Injection of C-SRC has little effect on development at the larval stage. In contrast, N1-SRC overexpression leads to reduced anterior development, including smaller eyes, and at higher doses, embryos exhibit axial shortening. Thus, our data support the proposal that N1-SRC–specific functions are determined by its SH3 domain insertion, which reduces binding affinity to ligands but increases intrinsic kinase activity.

## Discussion

### N1-SRC is a multilevel regulator of RNA metabolism

Compared to C-SRC, the downstream targets of N1-SRC are poorly characterized. Given that N1-SRC only differs from C-SRC by a small insertion in its SH3 domain, we investigated the mechanisms underpinning N1-SRC–specific functions by comparing the interactomes of the C-SRC and N1-SRC SH3 domains. Isolated SH3 domains have previously been used in high-throughput assays to successfully characterize targets of SRC family kinase regulation (Rickles et al., 1994; Sparks et al., 1996; Santoro et al., 1997). A caveat to this approach is that it may not recapitulate the binding specificity of full-length SRC kinases as it ignores the influence of other SRC protein domains in determining target specificity. The SH3 domain plays an important role in determining target specificity, but SH3 flanking sequences, the kinase domain, and intramolecular interactions with the SH2 domain also provide important context and affect SRC target specificity.

Perhaps surprisingly, we find that the N1-SRC SH3 insertion does not generate novel binding specificities but instead limits binding to a subset of C-SRC SH3 interacting proteins with lower affinity. In keeping with known SRC functions, C-SRC and N1-SRC SH3 domains interact with multiple proteins associated with the cytoskeleton, signal transduction, and membrane trafficking required for the terminal differentiation and function of neurons (Bjorge et al., 2000; Espada and Martín-Pérez, 2017; Koudelková et al., 2021). However, our analysis also reveals interactions with a subset of proteins involved in RNA metabolism, suggesting roles in splicing mRNA. Despite widespread regulation of splicing by serine/threonine protein kinases, such as SRPK, PKA, PKB, and GSK3β (reviewed by Stamm, 2008), the evidence for tyrosine kinase regulation is limited. C-SRC has been shown to regulate mRNA transport and processing, and this is dependent on the activity of the tyrosine kinase domain (Neel et al., 1995; Gondran and Dautry, 1999).

In support of the role of N1-SRC in regulating RNA metabolism, we show that mRNA splicing is altered in N1-SRC morphants, with exon skipping and intron retention being the two most frequent events detected. Prominent among the genes with altered splicing are *hnrnpa1* and *tra2a*, both of which encode RBPs. Thus, our data implicate N1-SRC as a multilevel regulator of RNA processing, not only interacting with RNA processing proteins but also involved in regulating the expression of RNA processing genes.

### SFPQ and FUS are candidate targets of N1-SRC phosphorylation

Our analysis of *Xenopus hnrnpa1* and *tra2a* splice junctions altered in N1-SRC morphants revealed the presence of multiple consensus sites for the SFPQ and FUS RBPs (Lerga et al., 2001; Ray et al., 2013), which have roles in the regulation of splicing in the nervous system (Orozco and Edbauer, 2013; Sama et al., 2014; Lim et al., 2020). Our analysis of public, experimentally derived phosphoproteomic datasets demonstrates that both SFPQ and FUS are targets of tyrosine phosphorylation by SRC family kinases and indicates that tyrosine phosphorylation of proteins involved in RNA metabolism is likely to be widespread. SFPQ is identified as an N1-SRC SH3 domain interactor, and conserved tyrosine phosphosites in SFPQ are phosphorylated by N1-SRC in vitro. Furthermore, C-terminal tyrosine phosphorylation of SFPQ/PSF has been shown to regulate its nuclear versus cytoplasmic localization, suggesting a mechanism by which N1-SRC might regulate SFPQ activity (Lukong et al., 2009).

FUS was not identified as a SRC SH3 domain interactor in our screen, but Y526 of FUS has previously been shown to be phosphorylated by SRC proteins. Y526 phosphorylation regulates stress-induced FUS aggregation and represents a possible mechanism for SRC regulation of FUS activity (Motaln et al., 2023). Although binding FUS and SFPQ at affected splice sites remains to be confirmed, we speculate that altered phosphorylation and activity of these splicing factors contributes to the observed changes in mRNA splicing in N1-SRC knockdown (morphant) embryos.

Data suggest that phosphorylation by N1-SRC is a likely mechanism for regulating the activity of splicing factors such as FUS and SFPQ. However, at present, the role of N1-SRC acting via kinase independent mechanisms, such as protein scaffolding, cannot be excluded (Brunton et al., 2005).

### Hnrnpa1 and Tra2 are candidate targets of the N1-SRC regulatory pathway

HNRNPA1 and TRA2A are RBPs with multiple roles in neural development and disease, and altered splicing events involving these proteins are the most significant splicing changes detected in N1-SRC morphant embryos.

HNRNPA1 is a multifunctional protein involved in the regulation of transcription, translation, mRNA transport, and mRNA splicing (Clarke et al., 2021). Altered patterns of splicing are associated with mislocalized HNRNPA1 protein in multiple sclerosis (Salapa et al., 2024). Transformer 2 RBPs belong to the serine-/arginine-rich (SR) family of splicing factors and were originally identified as regulators of mRNA splicing involved in *Drosophila* sex determination (Hoshijima et al., 1991). Vertebrates have two transformer 2 homologs, TRA2A and TRA2B, and there is emerging evidence that dysregulation of the human proteins is involved in abnormal patterns of splicing observed in some cancers (Xue et al., 2023). TRA2B knockdown in *Xenopus* results in abnormal splicing, including increased intron retention and exon skipping (Dichmann et al., 2015). Mice with a neuronal specific knock-out of TRA2B also have altered patterns of splicing and abnormal cortical development (Roberts et al., 2014; Storbeck et al., 2014).

Altered splicing of both *hnrnpa1* and *tra2a* in N1-SRC morphants affects the expression of their C-terminal GRDs. The HNRNPA1 GRD, known to mediate protein–protein and protein–RNA interactions (Chowdhury and Jin, 2023), is encoded by exons 6–9, and the retention of intron 7 truncates the GRD after exon 7. In TRA2A, the exon 6 skipping or intron 5/6 retention events lead to a loss of the GRD encoded within exon 6. GRDs are intrinsically disordered and are involved in phase separation and the formation of protein condensates (Lorkovic, 2012), and hence the loss or disruption of the HNRNPA1 and TRA2A GRDs by N1-SRC knockdown provides a mechanism for the downstream effects on neuronal gene expression we observed. Furthermore, regulation of the HNRNPA1 GRD by splicing is conserved in the human gene via a cassette exon. The HNRNPA1A and HNRNPA1B splice variants differ by the inclusion of 52 amino acids within the GRD of HNRNPA1B (Buvoli et al., 1990; Deshaies et al., 2018), close to the truncation site we observe following *Xenopus hnrnpa1* intron 7 retention. The physiological relevance of this splicing event is not entirely characterized, but it has recently been shown that HNRNPA1B is prone to aggregation (Deshaies et al., 2018). It should also be noted that the *Xenopus hnrnpa1* intron 7 retention event leads to the loss of the conserved “M9” domain encoded by exons 8 and 9, which is involved in regulating the nuclear import and export of HNRNPA1 (Michael et al., 1995) and points to a further mechanism by which the function of HNRNPA1 in neurogenesis is perturbed by N1-SRC knockdown.

### An N1-SRC regulated pathway during development and disease

Our data suggest novel roles of C-SRC and N1-SRC in the regulation of mRNA metabolism. Given that C-SRC expression is unaffected in N1-SRC morphant embryos and the overlapping binding specificities of their SH3 domains, why is C-SRC unable to substitute for N1-SRC in knockdown embryos? Our data suggest that while N1-SRC binds to a subset of C-SRC interacting proteins, the increased kinase activity of N1-SRC means that in the nervous system, these proteins are likely more highly phosphorylated than in regions where C-SRC alone is expressed, and this underpins the specific requirement for N1-SRC in neural development.

**Figure 7.**
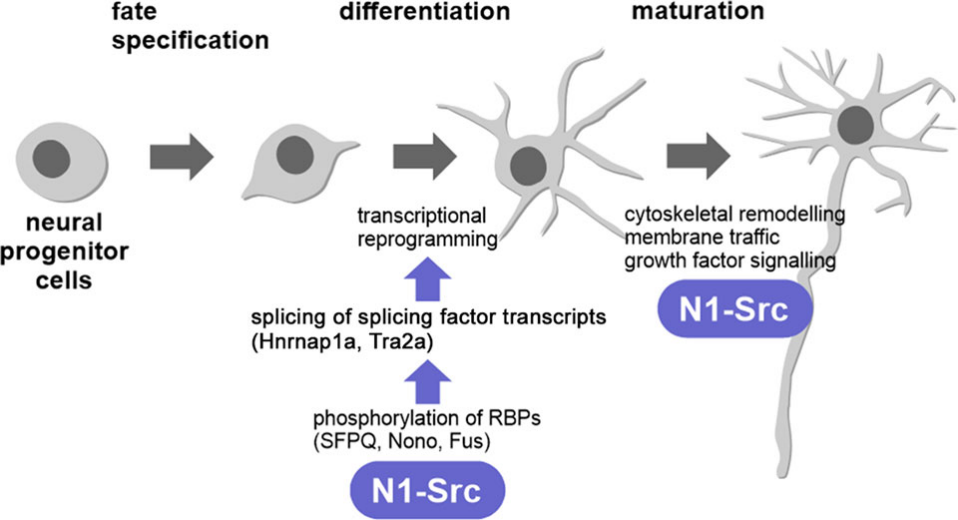
A model for N1-SRC function during neuronal specification and differentiation.

We propose a model in which N1-SRC kinase is required for neuronal differentiation by regulating the activity of RBPs, such as SFPQ and FUS, which in turn regulate splicing of a subset of mRNAs, including the splicing factors HNRNPA1 and TRA2A (Fig. 7). In this way N1-SRC contributes to the regulation of the transcriptional landscape necessary for neuronal differentiation. However, important questions remain, and the detailed mechanisms involved still remain unclear. In this regard, it will be important to investigate N1-SRC target interactions in vivo and how N1-SRC-mediated tyrosine-mediated phosphorylation might regulate the activity of splicing factors.

Our observations that N1-SRC function is required for mRNA splicing during neural development are of considerable interest because they support a novel paradigm for SRC family function that warrants investigation in other developmental and disease contexts. For example, high levels of N1- and N2-SRC expression are correlated with a positive prognosis in neuroblastoma (Bjelfman et al., 1990; Matsunaga et al., 1994), a developmental cancer of neural crest origin, in which splicing is altered (Shi et al., 2021). In connection with this, our GO analyses indicate GO terms associated with neural crest development are enriched in the cohort of genes downregulated in N1-SRC morphants (Extended Data Fig. 2-4). These data suggest that investigating the role of neuronal SRC isoforms in the regulation of splicing during neural crest, neurocristopathy, and neuroblastoma development is warranted.
