# Supplementary figures and images for: SRC Kinase Isoforms Regulate mRNA Splicing during Neural Development
Source: J Neurosci. 2025 Aug 1;45(34):e1705242025. doi: 10.1523/JNEUROSCI.1705-24.2025 (PMC12369926; doi:10.1523/JNEUROSCI.1705-24.2025)

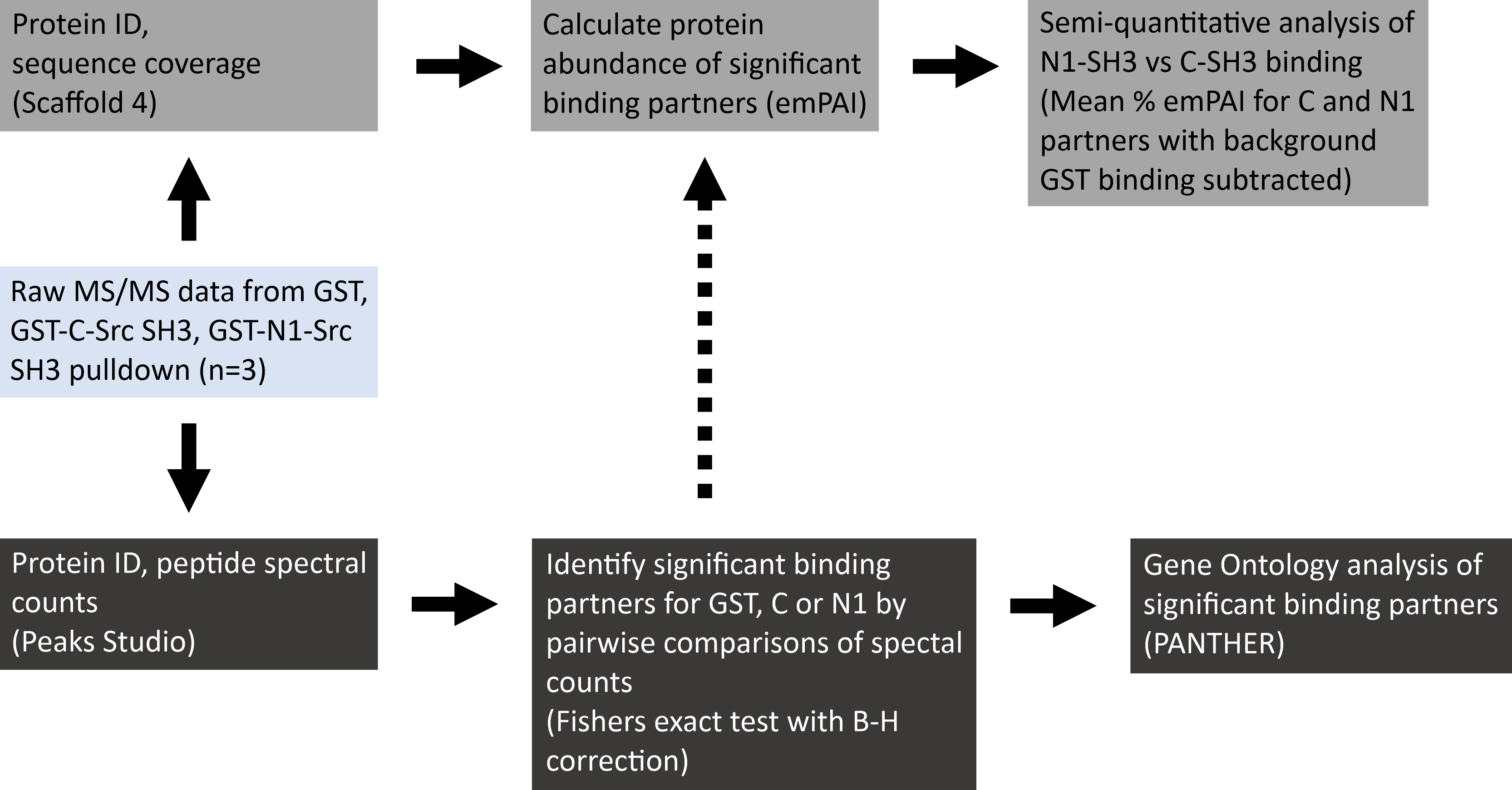

Supplement: Figure 1-1 — Proteomics analysis pipeline used in the study. Flow diagram summarising the approaches used to analyse the LC-MS/MS data arising from the C- and N1-SRC SH3 domain pulldown experiment. Download Figure 1-1, TIF file. [file jneuro-45-e1705242025-s001.tif]

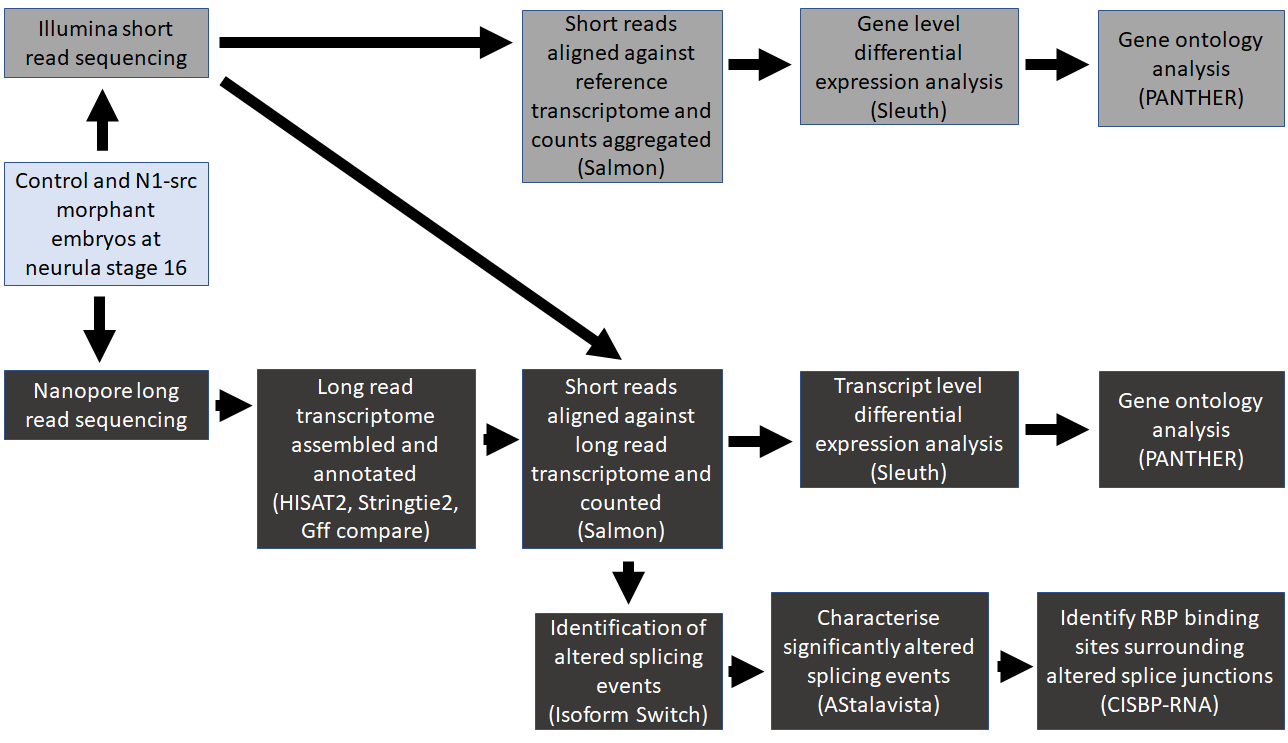

Supplement: Figure 2-1 — Transcriptomic analysis pipeline used in the study Flow diagram showing the multiple approaches used in this study to determine differential gene, transcript and splice variant expression in control and N1-SRC morphant embryos. Download Figure 2-1, TIF file. [file jneuro-45-e1705242025-s005.tif]

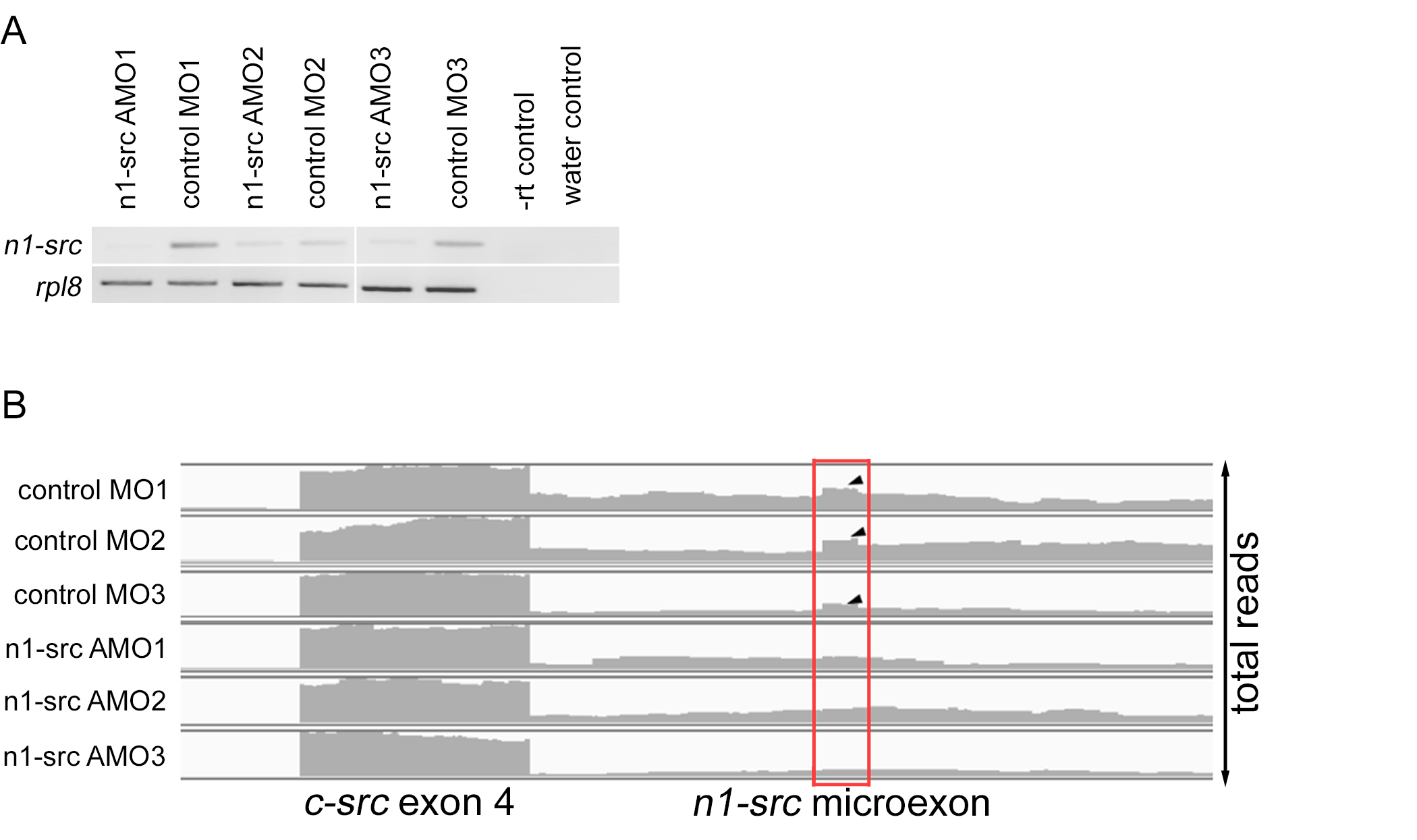

Supplement: Figure 2-2 — Injection of n1-src splice blocking antisense morpholinos effectively blocks N1-Src expression. A- rt-PCR analysis of n1-src expression in the three sets of neurula stage 16 control morpholino (control MO) injected and n1-src antisense, splice blocking morpholino (n1-src AMO) injected embryos used for RNA-Seq analysis. Rpl8 is used as a loading control. B- Mapping of Illumina short read sequences to n1-src microexon in control MO and n1-src AMO embryos. Download Figure 2-2, TIF file. [file jneuro-45-e1705242025-s006.tif]

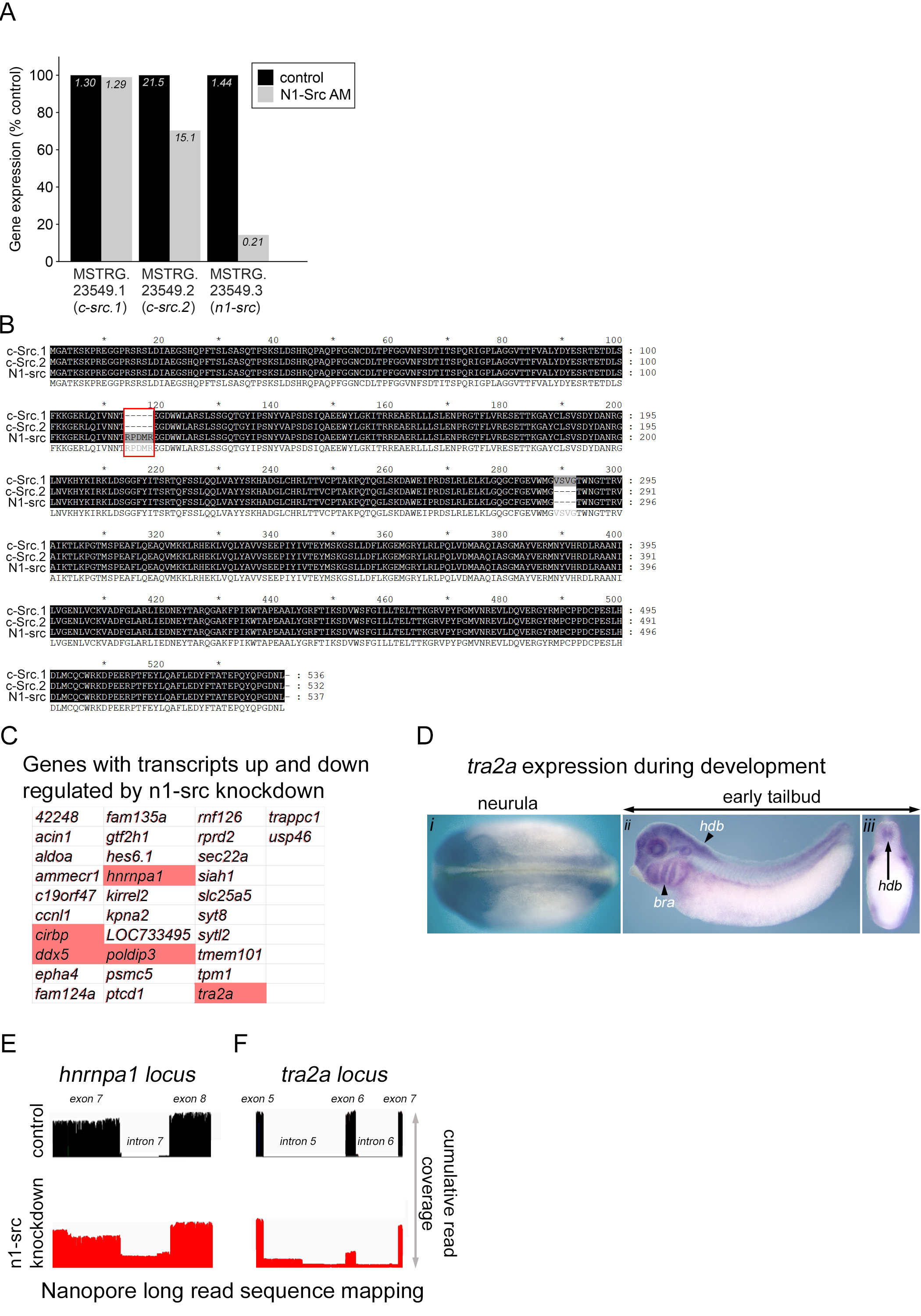

Supplement: Figure 4-3 — Altered splicing in N1-SRC knockdown embryos A- bar chart showing the relative expression of Src splice variants (c-src.1, c-src.2 and n1-src) in control and N1-SRC morphant embryos. Illumina sequencing reads were mapped to the Nanopore long read transcriptome and quantified using Salmon. Mean expression values in transcripts per million (TPM) calculated from Salmon transcriptome level output are included. B- conceptual peptide sequences of C-SRC.1, C-SRC.2 and N1-SRC splice variants. C- a list of genes which have transcripts both up and down regulated in n1-src knockdown embryos. Genes with functions associated with RNA metabolism and processing are shaded red. D- in situ hybridization analysis of tra2a expression during Xenopus development. (i) is a dorsal view of the neural plate of a neurula stage 16 embryo. (ii) is a lateral view (anterior to the left) of an early tailbud stage embryo, bra = branchial arch, hdb = hindbrain. E- Cumulative Nanopore long read sequences from control and n1-src knockdown embryos mapped to the region of retained intron 7 of hnrnpa1. F- Cumulative Nanopore long read sequences from control and n1-src knockdown embryos mapped to the region of skipped exon 6 and retained introns 5 and 6 of tra2a. Download Figure 4-3, TIF file. [file jneuro-45-e1705242025-s012.tif]

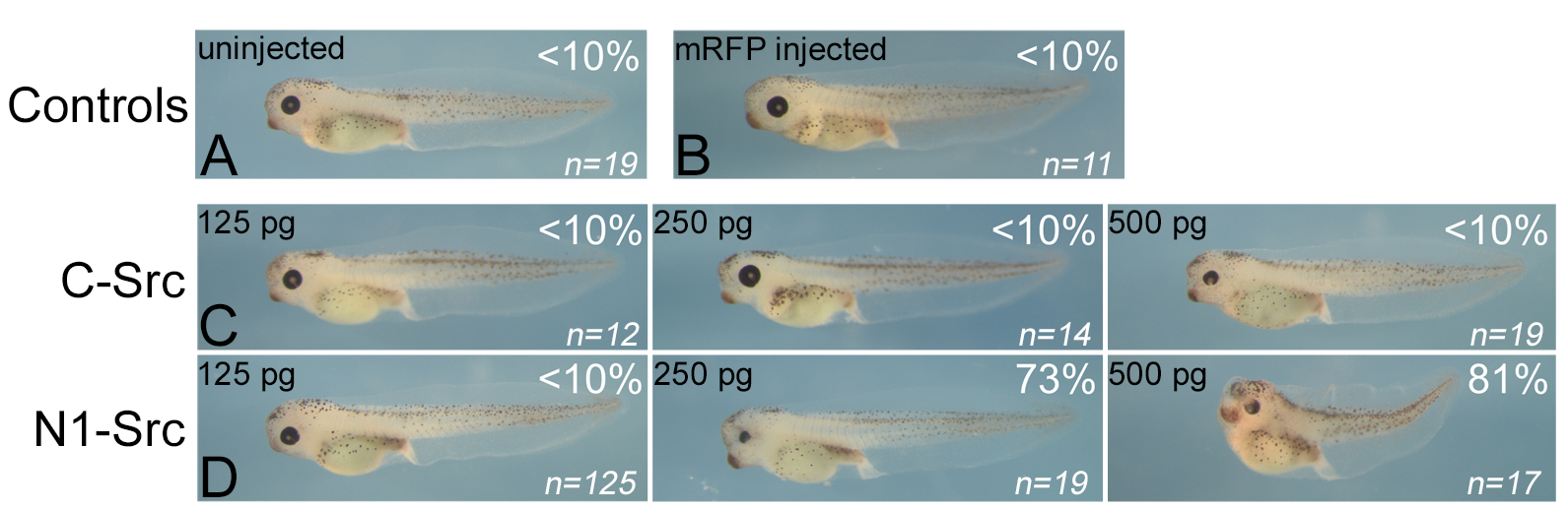

Supplement: Figure 6-2 — SRC and N1-SRC overexpression phenotypes in Xenopus embryos. Embryos were injected at the 2-cell stage and cultured to larval stage 41. N values and percentage of abnormal embryos are indicated in each panel. A- normal control uninjected embryo at larval stage 41. B- normal embryo injected with 500 pg synthetic monomeric red fluorescent protein mRNA (mRFP). C- representative embryos injected with 125 pg, 250 pg and 500 pg of synthetic C-SRC mRNA. Most embryos are normal but mild coloboma of the iris is present in the high dose. embryo presented. D- representative embryos injected with 125 pg, 250 pg and 500 pg of synthetic N1-SRC mRNA. The majority of embryos injected with >125 pg N1-SRC mRNA exhibit reduced development of the eyes and abnormal development of the main body, involving axial shortening and kinking. Download Figure 6-2, TIF file. [file jneuro-45-e1705242025-s018.tif]
